# ZIF-8-loaded decellularized porcine annulus fibrosus bioadhesive enhances rotator cuff tendon-to-bone healing in a rat model

**DOI:** 10.3389/fbioe.2025.1642818

**Published:** 2025-07-22

**Authors:** Xiping Jiang, Hui Xu, Xinyue Sun, Xuefan Yang, Yuxuan Xia, Wen Xue, Yaohua He

**Affiliations:** ^1^ Department of Orthopedic Surgery, Shanghai Sixth People’s Hospital Affiliated to Shanghai Jiao Tong University School of Medicine, Shanghai, China; ^2^ College of Biological Science and Medical Engineering, Donghua University, Shanghai, China; ^3^ Department of Dermatology, Renji Hospital, Shanghai Jiao Tong University School of Medicine, Shanghai, China; ^4^ Department of Orthopedic Surgery, Jinshan Branch of Shanghai Sixth People’s Hospital, Jinshan District Central Hospital Affiliated to Shanghai University of Medicine and Health Sciences, Shanghai, China

**Keywords:** rotator cuff, tendon-to-bone, decellularized extracellular matrix, bioadhesive, ZIF-8

## Abstract

**Introduction:**

The high rate of retear following rotator cuff repair is largely attributed to the absence of a fibrocartilage layer and limited bone regeneration capacity. We aim to evaluate a bioadhesive derived from decellularized porcine annulus fibrosus extracellular matrix, loaded with zeolitic imidazolate framework-8 (ZIF-8), and to promote rotator cuff tendon–bone healing.

**Methods:**

Three adhesive formulations were developed: (1) silk fibroin/tannic acid (ST group), (2) ST combined with decellularized porcine annulus fibrosus extracellular matrix (ST/dECM group), and (3) ST/dECM supplemented with ZIF-8 (ST/dECM/ZIF-8 group). Optimal component ratios were determined using lap shear strength testing. The microstructure, Fourier transform infrared (FTIR) spectra, swelling behavior, and degradation properties of the materials were characterized. *In vitro* studies assessed the adhesives’ effects on cytotoxicity, proliferation, and the chondrogenic and osteogenic differentiation of rat bone marrow-derived mesenchymal stem cells (BMSCs). A rat rotator cuff repair model was used to evaluate *in vivo* anti-inflammatory effects, fibrocartilage and bone regeneration, and biomechanical performance.

**Results:**

All adhesive formulations exhibited comparable tissue adhesion strength and biocompatibility. Both the ST/dECM and ST/dECM/ZIF-8 groups enhanced BMSC chondrogenic differentiation compared to the ST group, with the ST/dECM/ZIF-8 group showing superior osteogenic induction. *In vivo*, the ST/dECM/ZIF-8 hydrogel effectively reduced interfacial inflammation and promoted fibrocartilage and bone regeneration. Biomechanical testing demonstrated significantly higher ultimate load, tensile stress, and stiffness in all adhesive-treated groups compared to untreated controls.

**Conclusion:**

The ST/dECM/ZIF-8 bioadhesive hydrogel promotes fibrocartilage and bone regeneration. These findings highlight its potential as a promising biomaterial-based strategy to enhance tendon-to-bone interface healing following rotator cuff repair.

## 1 Introduction

The region where tendons attach to bone is known as the enthesis, a soft-to-hard tissue transition zone. This site is prone to injury and presents considerable challenges for repair. Rotator cuff injuries are among the most common shoulder injuries, with over 250,000 rotator cuff repair surgeries performed annually in the United States alone (Deprés-tremblay et al., 2016). However, despite continuous advancements in rotator cuff repair techniques, the postoperative re-tear rate remains high, ranging from 17% to 94% (Dimitrios et al., 2019; Jost et al., 2006), posing a major challenge to clinical outcomes.

Histologically, the rotator cuff tendon-to-bone interface typically consists of three functional regions: the tendon, fibrocartilage, and bone zone. This structural organization effectively alleviates mechanical stress concentration and facilitates a gradual load transfer between tendon and bone tissue (Zhang et al., 2012). However, after rotator cuff repair surgery, the native multi-layered functional structure of the interface is often replaced by fibrous scar tissue. Studies have shown that due to the inability to regenerate the original structure of the rotator cuff enthesis, the newly formed scar tissue demonstrates significantly inferior biomechanical properties compared to the natural interface, increasing the risk of postoperative retear (Song et al., 2024).

Based on the complex structure of the rotator cuff tendon-to-bone interface, promoting fibrocartilage regeneration is a key factor in mitigating stress concentration and reducing the high retear rate after rotator cuff repair. Biomimetic scaffolds derived from fibrocartilage-based decellularized extracellular matrix (dECM) have demonstrated potential in facilitating fibrocartilage regeneration at the enthesis. For instance, Tang et al. developed a layered scaffold using decellularized pubic symphysis matrix, which promoted the expression of cartilage-related genes in bone marrow-derived mesenchymal stem cells (BMSCs) *in vitro* and significantly enhanced fibrocartilage formation at the patellar enthesis in a rabbit model (Tang et al., 2020). Similarly, Olvera et al. immobilized decellularized articular cartilage matrix from the femoral head onto highly porous polycaprolactone (PCL) electrospun microfibers via covalent bonding or physical adsorption. BMSCs cultured on these scaffolds showed chondrogenic differentiation (Olvera et al., 2020). These studies suggest that decellularized fibrocartilage matrix can not only induce chondrogenic differentiation of stem cells *in vitro* but also regenerate functional fibrocartilage tissue *in vivo*.

The annulus fibrosus of the porcine intervertebral disc is also primarily composed of fibrocartilage. Previous studies have demonstrated that its decellularized matrix can significantly upregulate chondrogenic gene expression in stem cells *in vitro* (Liu et al., 2020; Liu C. et al., 2024). The annulus fibrosus is not a homogeneous structure. It can be subdivided into inner and outer regions based on cell type, matrix composition, and mechanical properties. The inner annulus fibrosus, adjacent to the nucleus pulposus, is rich in type II collagen and proteoglycans, with chondrocyte-like cells, whereas the outer annulus fibrosus contains spindle-shaped cells aligned along collagen fibers and secreting type I collagen (Liu et al., 2022). This compositional gradient is structurally similar to that of the rotator cuff enthesis, suggesting that annulus fibrosus-derived dECM holds significant potential as a biomimetic scaffold for reconstructing the fibrocartilaginous interface. Conventional dECM hydrogels based on collagen self-assembly suffer from long gelation times, poor mechanical strength, and inadequate adhesive properties in wet environments, limiting their application in ensuring stable fixation at the enthesis (Choi et al., 2024). To address this, we introduce silk fibroin and tannic acid as adhesive components to strengthen the fixation of fibrocartilage-derived dECM at the enthesis and amplify its regenerative efficacy.

Silk fibroin, a natural fibrous protein extracted from *Bombyx mori* cocoons, offers numerous advantages including excellent aqueous processability, abundant natural sources, facile functionalization, and low cost (Yuan et al., 2019). Tannic acid, a natural polyphenol found in various plants, exhibits antioxidant, anti-inflammatory, and anticancer activities, making it widely utilized in bioadhesive materials in recent years (Van Buren and Robinson, 1969; Wang et al., 2022). Rich in phenolic hydroxyl groups, tannic acid can form strong bonds with other molecules through dynamic interactions such as metal coordination, hydrogen bonding, and hydrophobic forces, thereby contributing to the development of high-performance functional materials (Nam et al., 2019). Silk fibroin molecules contain abundant carboxyl and hydroxyl groups, which can form stable complexes with the phenolic hydroxyl groups of tannic acid through hydrogen bonding and other non-covalent interactions (Gao et al., 2020). Adhesive materials based on the silk fibroin-tannic acid system have been applied across various fields. For example, Zou et al. fabricated a silk fibroin/tannic acid/Fe_3_O_4_ hydrogel by constructing a metal-phenolic network using tannic acid and Fe_3_O_4_ nanoparticles. This system exhibited fast gelation, shape adaptability, and strong adhesion, with good biocompatibility under static magnetic fields, and demonstrated excellent osteogenic effects both *in vitro* and *in vivo*, making it suitable for bone defect repair (Zou et al., 2023). Importantly, in the silk fibroin-tannic acid adhesive system, tannic acid not only contributes to strong adhesion but also plays a critical role in immunomodulation by regulating macrophage polarization. Numerous studies have shown that incorporating tannic acid into hydrogels enhances anti-inflammatory properties, creating a favorable microenvironment to support tissue regeneration (Wang et al., 2024; Zhang et al., 2024; Zhao et al., 2024).

In addition to fibrocartilage regeneration, bone regeneration is vital for functional healing at the enthesis (Killian et al., 2014). During rotator cuff repair, early-stage bone regeneration provides a stable anchoring point for tendon reattachment and is essential for preventing repair failure due to insufficient osseous support (Yakacki et al., 2010). To address this need, we incorporate zeolitic imidazolate framework-8 (ZIF-8) into the system. ZIF-8 is composed of tetrahedrally coordinated zinc ions and imidazolate ligands, combining the structural advantages of metal-organic framework (MOF) with the biological activity of zinc. On one hand, ZIF-8 can enhance the mechanical properties of the silk fibroin-tannic acid adhesive through metal coordination. On the other hand, the released zinc ions promote osteogenic differentiation of mesenchymal stem cells (MSCs) and facilitate bone tissue formation (Yang et al., 2022).

**FIGURE 1:**
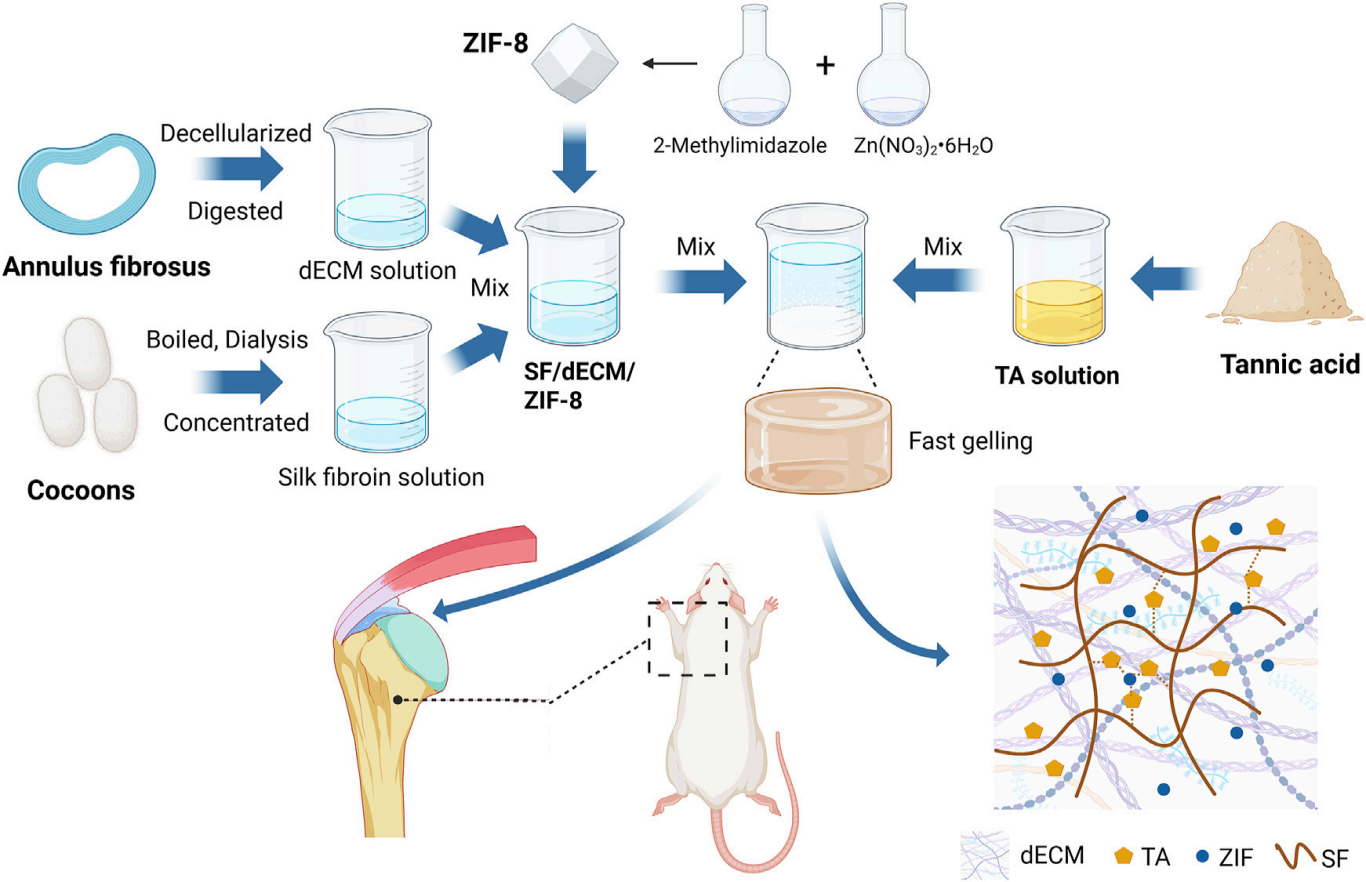
Schematic overview of the ZIF-8-loaded porcine annulus fibrosus-derived decellularized adhesive for rotator cuff tendon-to-bone interface regeneration in a rat model (W: weeks). Created with BioRender.com.

To overcome the limitations of current dECM hydrogels, such as weak adhesion and lack of immunomodulatory functionality. We propose a biomimetic hydrogel based on porcine annulus fibrosus-derived dECM, silk fibroin, and tannic acid, with the addition of ZIF-8 to impart osteoinductive capacity (Figure 1). In summary, this study aims to develop a decellularized adhesive scaffold that possesses good adhesive capability, immunomodulatory effects, fibrocartilage-mimicking composition, and bone regenerative potential, and to evaluate its efficacy in promoting tendon-to-bone interface healing following rotator cuff repair.

## 2 Materials and methods

### 2.1 Preparation and histological staining of decellularized matrix derived from porcine intervertebral disc annulus fibrosus

Fresh porcine intervertebral disc annulus fibrosus tissues were purchased from a local market. Decellularization was performed using a previously reported chemical-enzyme combined method (Liu B. et al., 2024). Fresh annulus fibrosus tissues were placed in 50 mM Tris buffer and stirred overnight. Tissues were treated with 1% Triton X-100 in Tris buffer at 4°C for 24 h, and digested in Hank’s balanced salt solution containing 40 U/mL DNase (Macklin Biochemical, Shanghai, China), 20 mg/L RNase (Solarbio Life Sciences, Beijing, China), and 0.01% trypsin (Sigma, NY, United States) at 37°C for 4 h. Samples were stirred overnight at 4°C in 1% Triton X-100/Tris buffer. After decellularization, the tissue samples were lyophilized and then stored at −20°C for later use. To prepare decellularized matrix solution, the lyophilized decellularized tissues were ground into powder in liquid nitrogen, and dissolved at 4°C in a 1 mg/mL pepsin solution prepared in 0.01 N HCl. The pH was then neutralized using 1 M NaOH solution. The prepared decellularized matrix solution was stored at 4°C for further use. For histological staining, both fresh and decellularized annulus fibrosus tissues were fixed in buffered formalin at 4°C for 2°days, then subjected to standard dehydration and paraffin embedding. The paraffin-embedded samples were sectioned at a thickness of 5 μm, and stained with hematoxylin and eosin (H&E) and Masson’s Trichrome.

### 2.2 Biochemical characterization of decellularized matrix derived from porcine intervertebral disc annulus fibrosus

The dry weight of the freeze-dried decellularized annulus fibrosus matrix was measured, and the tissue samples were then homogenized using a grinder. A 300 μg/mL papain solution (Meryer Biochemical Technology, Shanghai, China) was prepared using 50 mM phosphate buffer containing 5 mM L-cysteine (Meryer Biochemical Technology, Shanghai, China) and 5 mM ethylenediaminetetraacetic acid (EDTA) (Adamas Reagent, Shanghai, China), and the samples were digested at 65°C for 16 h.

Collagen content was determined using the hydroxyproline assay based on the above digestion solution, following the instructions of the hydroxyproline assay kit (Jiancheng Bioengineering Institute, Nanjing, China). Since hydroxyproline accounts for 13.4% of collagen, the collagen content was estimated accordingly. All collagen content results were normalized to tissue dry weight and expressed in μg/mg. Glycosaminoglycan (GAG) content was measured using the dimethylmethylene blue (1,9-dimethylmethylene blue chloride, DMMB) assay (Duan et al., 2015) based on the digestion solution. All GAG content results were normalized to tissue dry weight and expressed in μg/mg. DNA content was quantified using the PicroGreen double-stranded DNA assay (Yeasen Biotechnology, Shanghai, China) based on the above digestion solution. All DNA content results were normalized to tissue dry weight and expressed in ng/mg.

### 2.3 Preparation and characterization of ZIF-8

Dissolve zinc nitrate hexahydrate (Sigma, MO, United States) at 0.07 g/mL in 7.5 mL of deionized water, and 2-methylimidazole (Macklin, Shanghai, China) at 0.1 g/mL in 7.5 mL of methanol. Mix the solutions and stir at room temperature for 2 h. Separate the product by centrifugation, wash the precipitate with ethanol, and freeze-dry it. Store the powder at −20°C. Analyze it via scanning electron microscope (SEM, SU8010, Hitachi, Tokyo, Japan), energy dispersive X-ray spectroscopy (EDX), X-ray diffraction (XRD, D2 PHASE, Bruker, Karlsruhe, Germany), and fourier transform infrared spectrometer (FTIR, Tensor 27, Bruker, Karlsruhe, Germany). The ZIF-8 nanoparticle diameters in SEM images were measured using ImageJ.

### 2.4 Preparation of adhesives and tissue adhesion strength testing

Following the previously described method (Rockwood et al., 2011), a silk fibroin solution was prepared by degumming and dissolving silk fibers. Tannic acid powder (Aladdin, Shanghai, China) was dissolved in double-distilled water to prepare a 0.3 g/mL tannic acid solution. Mixed solutions were prepared using different concentrations of silk fibroin solution, decellularized matrix solution, and tannic acid solution, and varying amounts of ZIF-8. These compositions were added to formulate five groups of adhesives.

Pig skin was purchased from a local market. Adhesive samples from different groups were evenly applied between two pieces of pig skin tissue (dimensions: 30 mm × 5 mm) pre-wetted with phosphate buffered saline (PBS), with an overlapping area of 5 mm × 10 mm. After applying finger pressure for 15 s, a universal testing machine (1710-111, Instron, MA, United States) was used to perform lap shear testing. The test was conducted at a constant stretching speed of 10 mm/min until complete separation of the two tissue samples, and the maximum lap shear strength was recorded.

### 2.5 Micromorphology, FTIR, swelling and degradation characterization of adhesives

Based on the results of tensile testing, three groups of hydrogels were selected for subsequent experiments: (1) silk fibroin/tannic acid (ST group), (2) ST combined with decellularized porcine annulus fibrosus extracellular matrix (ST/dECM group), and (3) ST/dECM supplemented with ZIF-8 (ST/dECM/ZIF-8 group). The freeze-dried adhesives from these three groups were applied for SEM and FTIR analyses.

For each group, 200 μL of the hydrogel sample was prepared. The mass of each hydrogel adhesive was weighed and recorded as m (original). The samples were then immersed in 50 mL of PBS solution at room temperature. At time points of 0.5, 1, 2, 4, 20, and 24 h, surface liquid was blotted off, and the samples were weighed to record their swollen mass as m (swollen). The swelling ratio was calculated as:

$$\text{swelling ratio }\left(\%\right)=\frac{m\left(\text{swollen}\right)–\;m\left(\text{original}\right)}{m\left(\text{original}\right)}\times 100\%$$

Hydrogels made from ST, ST/dECM, and ST/dECM/ZIF-8 with a dry weight of 17–20 mg were weighed and recorded as W_0_. These samples were immersed in PBS solution at 37°C for 1, 3, 5, 7, and 14 days. At each time point, surface liquid was wiped off, and the dry weight of the hydrogel was measured and recorded as W_x_. The samples were then returned to the PBS solution for continued incubation. The degradation rate was calculated using the following formula:

$$d\text{egradation }r\text{ate }\left(\%\right)=\frac{W_{x}\;–\;W_{0}}{W_{0}}\times 100\%$$

### 2.6 Cytotoxicity and cell proliferation characterization of adhesive hydrogel extracts

Each group of prepared hydrogel samples was freeze-dried and ground into fine powder. The resulting powder was exposed to ultraviolet (UV) light for 24 h for sterilization. Then, 5 mg of the sample powder was weighed and added to 25 mL of complete culture medium, containing 1% penicillin/streptomycin (Hyclone, UT, United States), 10% fetal bovine serum (FBS, Cyagen Biosciences Inc., DE, United States), α-MEM culture medium (Hyclone, UT, United States). The mixture was incubated at 37°C for 24 h to obtain the extract, which was collected for subsequent experiments. The viability of passage 2 or 3 rat bone marrow mesenchymal stem cells (BMSCs, Cyagen Biosciences, DE, United States) was assessed after culturing in the extract-containing medium for 1, 2, and 3 days using the live/dead cell viability assay (Invitrogen, CA, United States) (Shi et al., 2021). Cell proliferation of passage 2 or 3 rat BMSCs cultured in the extract-containing medium for 1 and 3 days was evaluated using the MTT assay (Sigma, MO, United States) (Kong et al., 2021).

### 2.7 Chondrogenic and osteogenic differentiation of rat BMSCs

For chondrogenic differentiation, passage 2 or 3 rat BMSCs were co-cultured with adhesive hydrogels from different groups in chondrogenic differentiation medium and induced for 14 days. The chondrogenic differentiation medium consisted of: DMEM/F12 (Hyclone, UT, United States), 10% fetal bovine serum, 1% penicillin/streptomycin, 100 nM dexamethasone (Sigma, MO, United States), 0.2 mM ascorbic acid (Sigma, MO, United States), 1 mM sodium pyruvate (Sigma, MO, United States), insulin-transferrin-selenium (BD Biosciences, NJ, United States), and 10 ng/mL TGF-β3 (PetroTech, OK, United States) (Bai et al., 2022). For osteogenic differentiation, passage 2 or 3 rat BMSCs were cultured in osteogenic differentiation medium containing extracts of adhesive hydrogels from different groups and induced for 14 days (powder-to-medium mass/volume ratio = 5 mg: 25 mL). The osteogenic differentiation medium consisted of: DMEM/F12, 10% fetal bovine serum, 1% penicillin/streptomycin, 100 nM dexamethasone, 10 mM β-glycerophosphate (Sigma, MO, United States), and 50 μM ascorbic acid (Ogawa et al., 2004). Total RNA was extracted from rat BMSCs cultured in differentiation medium for 14 days using an RNA extraction kit (Servicebio, Hubei, China). The concentration and purity of the RNA were measured using a NanoDrop spectrophotometer (Thermofisher, DE, United States). Qualified RNA samples were immediately subjected to reverse transcription. cDNA was synthesized from the total RNA using a cDNA synthesis kit (Servicebio, Hubei, China), and quantitative real-time PCR (qPCR) was performed using SYBR Green (Servicebio, Hubei, China) on a real-time PCR system. GAPDH was used as the internal reference gene to normalize the expression levels of target genes. The relative expression of genes related to chondrogenic and osteogenic differentiation was analyzed using the comparative Ct (2^−ΔΔCT^) method. All primers are shown in Supplementary Table S1.

### 2.8 Cell immunofluorescence staining

Passage 2 or 3 rat BMSCs were cultured with different adhesives in chondrogenic differentiation medium and on the slides. The slides were washed with PBS. A 3% bovine serum albumin (BSA) solution was added to the reaction area to fully cover the sample, and the sample was incubated at room temperature. After discarding the blocking solution, primary antibody working solution against aggrecan (13,880-1 AP, ProteinTech, Hubei, China) was added. The samples were transferred to a culture chamber and incubated at 4°C for 16–18 h. Following incubation, the samples were incubated with a CY3-labeled goat anti-rabbit IgG secondary antibody (111-165-003, Jackson, MS, United States) at room temperature. After additional PBS washes, DAPI staining solution was added for nuclear staining. Coverslips were mounted using anti-fade mounting medium. The prepared samples were immediately imaged using a laser confocal microscope (Leica, Wetzlar, Germany). Semi-quantitative analysis of immunofluorescence intensity was performed using ImageJ software.

### 2.9 Alkaline phosphatase (ALP) staining and activity assay

Passage 2 or 3 rat BMSCs were cultured with different adhesive extracts in osteogenic differentiation medium. After removing the culture medium, the samples were washed with PBS and then fixed with 4% paraformaldehyde (PFA) at room temperature. After fixation, the samples were washed again with PBS. The ALP staining kit (Beyotime, Shanghai, China) was applied. A chromogenic working solution was prepared by mixing BCIP stock solution, NBT stock solution, and ALP chromogenic buffer. The samples were covered with the staining solution and incubated at room temperature. Microscopic imaging was performed using a light microscope. The ALP activity assay kit (Beyotime, Shanghai, China) was applied. The chromogenic substrate was prepared, and p-nitrophenol standards were serially diluted to a working concentration. After removing the culture medium, RIPA lysis buffer (Beyotime, Shanghai, China) was added to each well, and the supernatant was collected for analysis. Gradient volumes of the standard solution and different testing samples were prepared and measured following the kit. Enzyme activity was calculated based on the standard curve, expressed in units of enzyme activity within the diethanolamine (DEA) buffer system. Total protein concentration of the samples was also determined, and the final result was presented as enzyme activity units per protein concentration.

### 2.10 Animal experiments

The animal experiments were approved by the institutional animal care and use committee of Shanghai Sixth People’s Hospital affiliated to Shanghai Jiao Tong University School of Medicine (Approval No.: DWLL2025-0818). A total of 40 male Sprague-Dawley (SD) rats (weighing 250–300 g) underwent rotator cuff injury and repair surgery, as detailed below: Anesthesia was induced using 2%–3% isoflurane (Reward life science, Guangdong, China) delivered via an animal gas anesthesia system (EZSystem, CA, United States). After skin incision, the distal clavicle was located, and the coracoclavicular ligament was severed to expose the supraspinatus tendon insertion site (Supplementary Figure S2A,D). The tendon was detached from the humeral insertion using a scalpel, and the tendon-to-bone insertion site was carefully debrided. A bone tunnel was created in the humerus using an electric drill, and a 4-0 suture was passed through the tunnel. According to experimental group assignment, 200 μL of the corresponding adhesive hydrogel was implanted between the tendon and bone (Supplementary Figure S2B,E). The sutures were then passed through the torn end of the supraspinatus tendon and tied beneath the bone tunnel to secure the repair (Supplementary Figure S2C,F). The skin was closed using 3-0 sutures, and penicillin was administered postoperatively to prevent infection. The rats were divided into four groups. Defect group: rotator cuff injury and repair surgery without hydrogel implantation; ST group: repair surgery with implantation of ST group hydrogel; ST/dECM group: repair surgery with implantation of ST/dECM hydrogel; ST/dECM/ZIF-8 group: repair surgery with implantation of ST/dECM/ZIF-8 hydrogel. At 4 weeks post-surgery, 16 rats (4 per group) were euthanized for tissue collection and histological staining. At 8 weeks post-surgery, the remaining 24 rats (6 per group) were euthanized for histological staining, micro-CT analysis, and biomechanical testing of the humerus-supraspinatus complex (Supplementary Figure S2G).

### 2.11 Harvesting of the tendon-bone unit and histological staining analysis

The humerus-supraspinatus tendon units were dissected from the rats (Supplementary Figure S4). The samples were fixed in a universal tissue fixative, followed by decalcification, graded ethanol dehydration, and paraffin embedding. Longitudinal sections (5 μm thick) were cut along the long axis of the supraspinatus tendon and subjected to H&E and Masson’s trichrome staining. Tendon-to-bone healing at the reconstructed supraspinatus tendon insertion was evaluated using the tendon maturation scoring system proposed by Ide et al. (Ide et al., 2009). Histological images were reviewed in a blinded manner by two histologists. Additional sections of the humerus-supraspinatus tendon unit were also prepared for safranin O and picrosirius red staining analysis. For picrosirius red staining, images were obtained with a polarized microscope (DS-Ri2, Nikon, Tokyo, Japan). Five randomly selected areas underwent quantitative assessment using gray scale values. These values correspond to collagen birefringence against a dark background, with higher values correlating with greater collagen fiber maturity (Jiang et al., 2022). Semiquantitative analyses were performed using ImageJ software.

### 2.12 Immunofluorescence staining

Tissue sections were sequentially immersed in xylene, followed by dehydration through a graded anhydrous ethanol series, and then rinsed with deionized water. Antigen retrieval was performed using EDTA buffer in a microwave oven. After cooling, 3% hydrogen peroxide solution was applied and incubated in the dark at room temperature. After washing with PBS, 3% BSA blocking solution was applied and incubated at room temperature. The tissue sections were then incubated overnight at 4°C in a humid chamber with diluted primary antibody iNOS (HA722031, Huabio, Zhejiang, China) and CD206 (ab300621, Abcam, MA, United States) solution. After PBS washing, species-matched secondary antibodies conjugated with horseradish peroxidase (ab205718, Abcam, MA, United States) were applied and incubated at room temperature. DAPI staining solution was added and incubated in the dark, followed by PBS washes. Finally, the sections were sealed with an anti-fade mounting medium. Fluorescence images were acquired using a laser confocal microscope.

### 2.13 Micro-CT analysis

The humerus-supraspinatus units were harvested from the rats and fixed in a universal tissue fixative to preserve tissue integrity. The samples were then scanned using a micro-CT scanner (nanoVoxel-2000, Sanying Precision Engineering, Tianjin, China). Scanning parameters were set as follows: X-ray source voltage at 85 kV, current at 100 μA, 360° rotation with 720 frames at 0.5° per frame, and a scan resolution of VoxelDis = 9.99 μm. Images were acquired using NanoVoxel Scan software version 2.1.702.0 (Sanying Precision Engineering, Tianjin, China), and three-dimensional reconstruction was performed using VoxelStudio Recon software version 2.5.1.25 (Sanying Precision Engineering, Tianjin, China). Image analysis was conducted using CT Vox software version 3.2 (Bruker, Karlsruhe, Germany). The region of interest (ROI) was the rotator cuff bone tunnel, and the analyzed parameters included bone volume fraction (BV/TV) and trabecular number (Tb.N).

### 2.14 Biomechanical testing of the tendon-bone unit

The humerus of the experimental animals was fixed in a 1.5 mL centrifuge tube using hot-melt adhesive, ensuring full exposure of the articular surface of the humeral head. A scalpel was used to carefully dissect along the direction of the supraspinatus muscle fibers, preserving the integrity of the tendon-to-bone interface structure. The tendon was secured using sandpaper to ensure stability during testing. The attachment area of the tendon was measured using a vernier caliper. Biomechanical testing was conducted using a mechanical testing machine (HSS-DX1000, Heng Rui Jin Testing Machine Co., Ltd., Shandong, China). Each sample was initially preloaded with a tensile force of 0.1 N, followed by loading at a constant rate of 10 mm/min until failure. The load-displacement curve was recorded, and stiffness was calculated accordingly. The maximum load was directly obtained from the testing machine, and the ultimate stress was calculated by dividing the ultimate load at failure by the cross-sectional area of the tendon.

### 2.15 Statistical analysis

The data for DNA, collagen, and GAG content in the decellularized matrix were analyzed using an unpaired t-test. One-way analysis of variance (one-way ANOVA) followed by Tukey’s *post hoc* test was used for analyzing lap shear test mechanical properties, swelling ratio and degradation rate at different time points, qPCR results, MTT assay, semi-quantitative analysis of immunofluorescence intensity, ALP activity assay, Safranin O staining heterochromia, micro-CT results, and biomechanical testing data from *ex vivo* rat tendon-bone unit experiments. Histological scores were compared using the Kruskal-Wallis test. All statistical analyses were performed using GraphPad Prism version 9.0.0. Significance levels were indicated: no significance (ns), *p < 0.05, **p < 0.01, ***p < 0.001.

## 3 Results

### 3.1 Histological staining and biochemical composition analysis of dECM derived from porcine intervertebral disc annulus fibrosus

**FIGURE 2:**
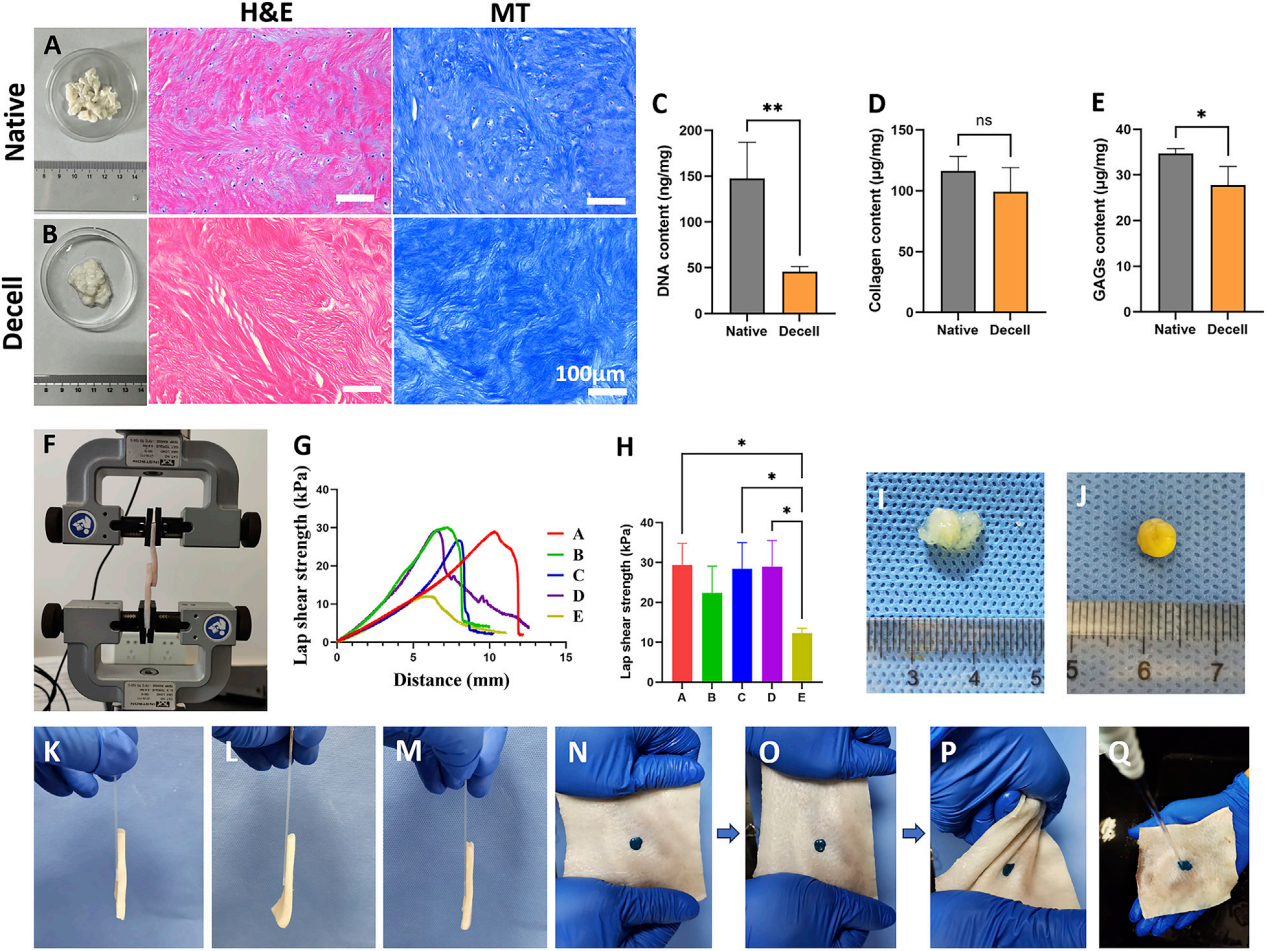
The histological and biochemical characteristics of decellularized extracellular matrix and tissue adhesion strength of different adhesives. **(A,B)** Representative **(H,E)** and Masson’s Trichrome Staining of native **(A)** and decellularized **(B)** extracellular matrix. The scale bars for the H&E staining and Masson’s trichrome staining are consistent. **(C–E)** The DNA **(C)**, collagen **(D)**, and GAG **(E)** contents of native and decellularized extracellular matrix (n = 4). **(F)** Schematic diagram of tissue adhesion strength testing using porcine skin. **(G)** Representative tensile curves of adhesives with different compositions. **(H)** Test results of tissue adhesion strength for adhesives with different compositions (n = 3). **(I)** Direct reaction of tannic acid and decellularized matrix failed to form a stable gel. **(J)** Gel formation was achieved by reacting tannic acid with a decellularized matrix supplemented with silk fibroin. **(K)** Adhesive from the SF group adhered porcine skin to a glass slide. **(L)** Adhesive from the DC group adhered porcine skin to a glass slide. **(M)** Adhesive from the ST/dECM/ZIF-8 group adhered porcine skin to a glass slide. **(N)** ST/dECM/ZIF-8 group adhesive stained with green food dye adhering to porcine skin. **(O)** Tensile testing performed on porcine skin. **(P)** Torsion testing performed on porcine skin. **(Q)** Water flow impact testing performed on porcine skin.

H&E and Masson’s trichrome staining were used to compare the porcine intervertebral disc annulus fibrosus tissue before and after decellularization (Figures 2A,B). The results indicated that the original nuclear structures had largely disappeared following decellularization, indicating successful removal of cellular components. Moreover, the tissue morphology of the annulus fibrosus was well preserved without extensive structural damage. To further quantitatively assess changes in extracellular matrix components after decellularization, biochemical assays were performed to measure the contents of DNA, collagen, and GAGs before and after treatment. The DNA content significantly decreased after decellularization (Figure 2C), meeting the standard for decellularized materials (less than 50 ng/mg). The collagen content in the decellularized annulus fibrosus remained comparable to that of the untreated group (Figure 2D). Although the GAG content in the decellularized group was reduced compared to the untreated group, it remained at a relatively high level overall (Figure 2E).

### 3.2 Material characterization of ZIF-8

The synthesized ZIF-8 was characterized using SEM and EDX for elemental mapping. The diameter of ZIF-8 particles was approximately 80 nm. The EDX spectrum revealed characteristic peaks for Zn and N elements, corresponding to the metallic and organic components of the ZIF-8 ligand, respectively (Supplementary Figure S1A). Supplementary Figure S1B displays the XRD pattern of ZIF-8. The diffraction peak at 7.6° corresponds to the [011] plane, representing the most intense peak of ZIF-8 and indicating diffraction from a densely packed plane in a cubic crystal system. The peak at 10.6° corresponds to the [002] plane, a characteristic reflection of the cubic structure, indicating periodic arrangement along the c-axis. The 13.0° peak corresponds to the [112] plane, a higher-order diffraction peak that further confirms the cubic symmetry. The peak at 18.2° corresponds to the [222] plane, reflecting smaller interplanar spacing (Zhou et al., 2017). Additionally, the FTIR spectrum shown in Supplementary Figure S1C reveals an absorption peak at 1,585 cm^-1^ attributed to C=N stretching vibration of the imidazole ring, a peak at 1,144 cm^-1^ corresponding to C–N stretching vibration, and a peak at 420 cm^-1^ associated with the Zn–N coordination bond vibration (Maghsoudi et al., 2024).

### 3.3 Comparison of mechanical properties of hydrogels with different formulations

According to previous reports, in silk fibroin-tannic acid adhesives, the concentration of silk fibroin solution typically ranges from 5 wt% to 10 wt%, and tannic acid solution ranges from 0.1 g/mL to 0.6 g/mL (Luo et al., 2020; Guo et al., 2024; Kim et al., 2023). Since the silk fibroin solution would be further diluted in subsequent experiments, a higher concentration of 10 wt% silk fibroin solution was selected for this study. Additionally, a 0.3 g/mL tannic acid solution was chosen because its pH is close to the theoretical isoelectric point of silk fibroin, which facilitates rapid gelation through a dual mechanism: suppressing electrostatic repulsion between molecules and inducing hierarchical and orderly assembly of adjacent silk fibroin chains (Zhang et al., 2011). Based on previous literature, cartilage-derived dECM, used to induce chondrogenic differentiation of mesenchymal stem cells, is typically applied at concentrations between 1 wt% and 5 wt%. In this study, concentrations of 1.25 wt% and 2.5 wt% were selected for evaluation (Zeng et al., 2022; Li et al., 2024). ZIF-8 was chosen at 0.5 wt% and 1 wt% based on prior findings that indicate its biosafety and osteoinductive properties within certain concentration ranges (Tang et al., 2024).

To assess the wet-state tissue adhesive strength, five adhesive formulations were tested using a universal testing machine (Figure 2F). The five groups were formulated as follows: group A: 10 wt% silk fibroin solution and 0.3 g/mL tannic acid solution mixed at a volume ratio of 1:1; group B: 10 wt% silk fibroin solution, 1.25 wt% dECM solution, and 0.3 g/mL tannic acid solution mixed at a volume ratio of 2:1:2; group C: 10 wt% silk fibroin solution, 2.5 wt% dECM solution, and 0.3 g/mL tannic acid solution mixed at a volume ratio of 2:1:2; group D: 10 wt% silk fibroin and 2.5 wt% dECM mixture containing 0.5 wt% ZIF-8, mixed with 0.3 g/mL tannic acid solution at a volume ratio of 2:1:2; group E: 10 wt% silk fibroin and 2.5 wt% dECM mixture containing 1 wt% ZIF-8, mixed with 0.3 g/mL tannic acid solution at a volume ratio of 2:1:2. Figure 2G shows the representative tensile strength curves for groups A-E. The addition of dECM at different concentrations (groups B and C) to the base formulation (group A) did not significantly affect the adhesive mechanical strength (Figure 2H). Since a higher concentration of dECM is more favorable for chondrogenic differentiation of mesenchymal stem cells, group C was selected for further experiments. Based on the composition of group C, different amounts of ZIF-8 were added (groups D and E). The results showed that 0.5 wt% ZIF-8 (group D) did not negatively affect mechanical performance, whereas 1 wt% ZIF-8 (group E) led to a reduction in mechanical strength. Therefore, group D was chosen for subsequent studies. Based on the tissue adhesion testing results, three formulations were selected for further material, cellular, and animal experiments. They were ST group: 10 wt% silk fibroin solution and 0.3 g/mL tannic acid solution mixed at a 1:1 volume ratio; ST/dECM group: 10 wt% silk fibroin solution, 2.5 wt% dECM solution, and 0.3 g/mL tannic acid solution mixed at a 2:1:2 volume ratio; ST/dECM/ZIF-8 group: 10 wt% silk fibroin solution containing 0.5 wt% ZIF-8, 2.5 wt% dECM solution, and 0.3 g/mL tannic acid solution mixed at a 2:1:2 volume ratio.

As shown in Figure 2I, adhesives prepared using only tannic acid and dECM exhibited poor gelation and insufficient adhesive strength and were therefore excluded from further investigation. However, adding silk fibroin to the dECM prior to mixing with tannic acid significantly improved gelation performance (Figure 2J). Figures 2K–M show the adhesion performance of ST, ST/dECM, and ST/dECM/ZIF-8 adhesives in bonding porcine skin directly to a glass slide. In the ST/dECM/ZIF-8 group, green food dye was added to the adhesive and applied to the porcine skin (Figure 2N). Following mechanical manipulation such as stretching (Figure 2O) and twisting (Figure 2P) of the porcine skin, the ST/dECM/ZIF-8 adhesive demonstrated excellent adhesion. Furthermore, the ST/dECM/ZIF-8 adhesive remained well-attached to the porcine skin even under running water, indicating its strong tissue adhesion in dynamic wet environments (Figure 2Q).

### 3.4 SEM characterization, FTIR analysis, swelling ratio, and degradation rate of hydrogels with different formulations

**FIGURE 3:**
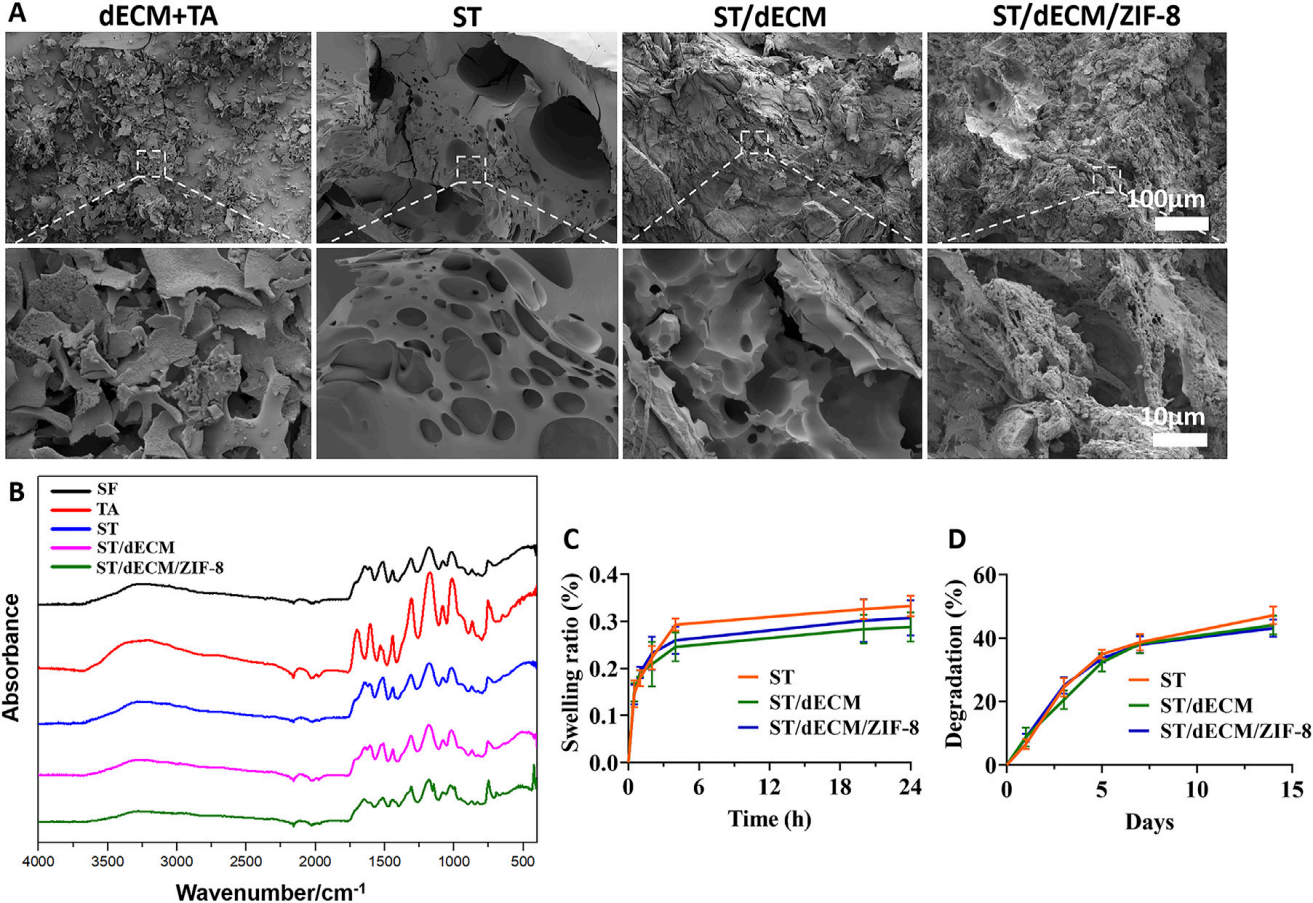
The SEM images, FTIR, swelling ratio, and degradation of hydrogels with different formulations. **(A)** SEM images. **(B)** FTIR results. **(C)** Swelling ratio (n = 3). **(D)** Degradation of adhesive hydrogels with different formulations (n = 3). The dECM + TA group consists of a mixture of decellularized extracellular matrix (dECM) solution and tannic acid (TA). SF refers to silk fibroin, TA to tannic acid, ST is the hydrogel formed by silk fibroin and tannic acid, ST/dECM is the hydrogel formed by silk fibroin (with added decellularized extracellular matrix, dECM) and tannic acid, and ST/dECM/ZIF-8 is the hydrogel formed by silk fibroin (with added dECM) and tannic acid, with the addition of ZIF-8.

The SEM results showed that the mixture of dECM and tannic acid alone failed to form a well-structured gel, whereas the other three formulations are able to form relatively dense hydrogel structures (Figure 3A). According to the FTIR spectra of the samples (Figure 3B), the shared broad peak at 3,276–3,291 cm^-1^ corresponded to O–H and N–H stretching vibrations, indicating the presence of abundant hydrogen bonds in all samples. The common peak at 1,445 cm^-1^ was attributed to CH_2_/CH_3_ bending or aromatic C=C stretching vibrations, typically found in proteins and phenolic compounds. Characteristic peaks of silk fibroin included 1,656 cm^-1^ (amide I band), associated with C=O stretching in β-sheet structures; 1,512 cm^-1^ (amide II band), attributed to N–H bending and C–N stretching; 1,313 cm^-1^ (amide III band), associated with C–N and N–H vibrations reflecting secondary structural changes; and 1,082 cm^-1^ and 1,016 cm^-1^, corresponding to C–O vibrations. For tannic acid, the characteristic peak at 1700 cm^-1^ was due to C=O stretching, while peaks at 1,607 cm^-1^ and 1,534 cm^-1^ corresponded to C=C stretching in aromatic rings, and 1,175 cm^-1^ was due to C–O stretching in phenolic hydroxyl groups. In the ST, ST/dECM, and ST/dECM/ZIF-8 composite hydrogels, the characteristic tannic acid peak at 1700 cm^-1^ disappeared or was weakened, likely due to hydrogen bonding or ionic interactions between ester or carboxyl groups in tannic acid and amino groups in silk fibroin, resulting in shifts or reductions in C=O peak intensity. No significant changes in the FTIR spectra were observed before and after the addition of dECM, possibly because the dECM content was low compared to silk fibroin and its major components were similar to those of silk fibroin, leading to overlapping peaks. In the ST/dECM/ZIF-8 composite hydrogel, new peaks appeared: 1,149 cm^-1^ corresponded to C–N stretching in the imidazole ring of ZIF-8, 998 cm^-1^ might be attributed to Zn–N coordination or imidazole ring deformation in ZIF-8, and 696 cm^-1^ corresponded to out-of-plane bending of the imidazole ring, indicating successful incorporation of ZIF-8. These results suggested that hydrogen bonding, coordination interactions, and hydrophobic interactions played a dominant role in forming the multicomponent composite system, and the inclusion of ZIF-8 further enhanced the crosslinked network via metal coordination.

After 24 h, the swelling ratios of the three adhesives were 33.24% ± 2.17%, 28.83% ± 3.12%, and 30.74% ± 3.71%, respectively (Figure 3C). After 14 days, their degradation rates were 47.17% ± 2.78%, 44.12% ± 2.94%, and 43.15% ± 2.74%, respectively (Figure 3D). The three bioadhesives exhibited similar swelling and degradation behavior, indicating that the addition of dECM and ZIF-8 had no significant impact on the swelling or degradation properties of the adhesives.

### 3.5 Cytocompatibility and effects of adhesives with different formulations on proliferation, chondrogenic and osteogenic differentiation of rat BMSCs

**FIGURE 4:**
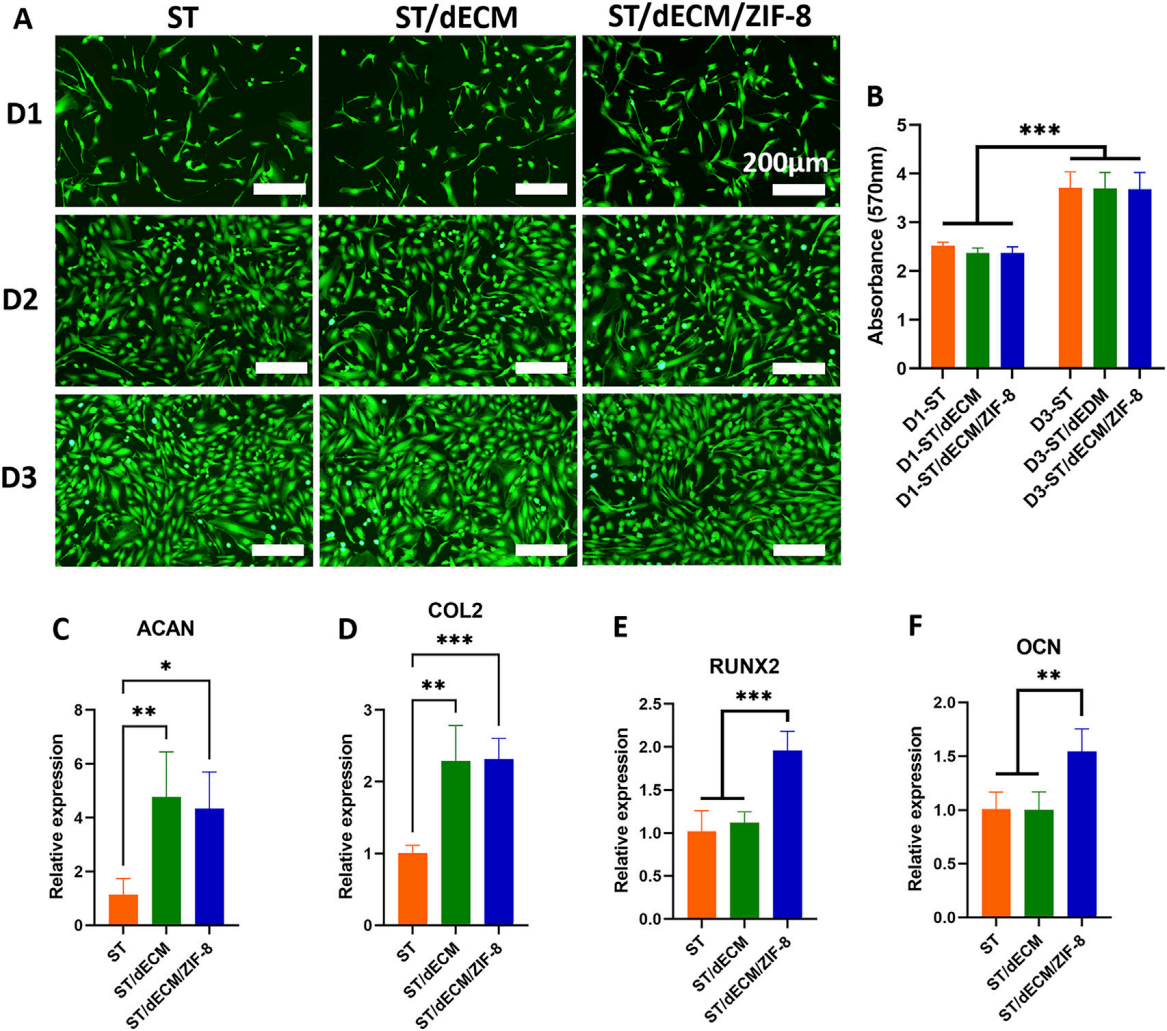
Effects of different adhesives extracts on cytocompatibility, cell proliferation, and chondrogenic and osteogenic differentiations of rat BMSCs. **(A)** Live/dead staining images of rat BMSCs cultured in different adhesive extracts (ST, ST/dECM, and ST/dECM/ZIF-8 groups) at days 1, 2, and 3 (D1, D2, D3) (scale bar = 200 μm). **(B)** Proliferation activity of rat BMSCs treated with extracts from ST, ST/dECM, and ST/dECM/ZIF-8 adhesive groups (n = 4). **(C,D)** The effect of co-culturing rat BMSCs with different formulation adhesives on the expression of chondrogenic differentiation-related genes (n = 4). ACAN **(C)**, and COL2 **(D)** expression of rat BMSCs cultured with different adhesive hydrogel extracts. **(E,F)** The gene expression of rat BMSCs cultured with adhesive hydrogel extracts with different formulations (n = 4). RUNX2 **(E)**, OCN **(F)** expression of rat BMSCs cultured with different adhesive hydrogel extracts.

Co-culture of rat BMSCs with the extracts of ST, ST/dECM, and ST/dECM/ZIF-8 adhesives demonstrated good cytocompatibility on days 1, 2, and 3 of culture (Figure 4A). The MTT semi-quantitative assay results indicated that rat BMSCs proliferated in all three adhesive extracts, with no significant differences in proliferation trends among the groups (Figure 4B).

**FIGURE 5:**
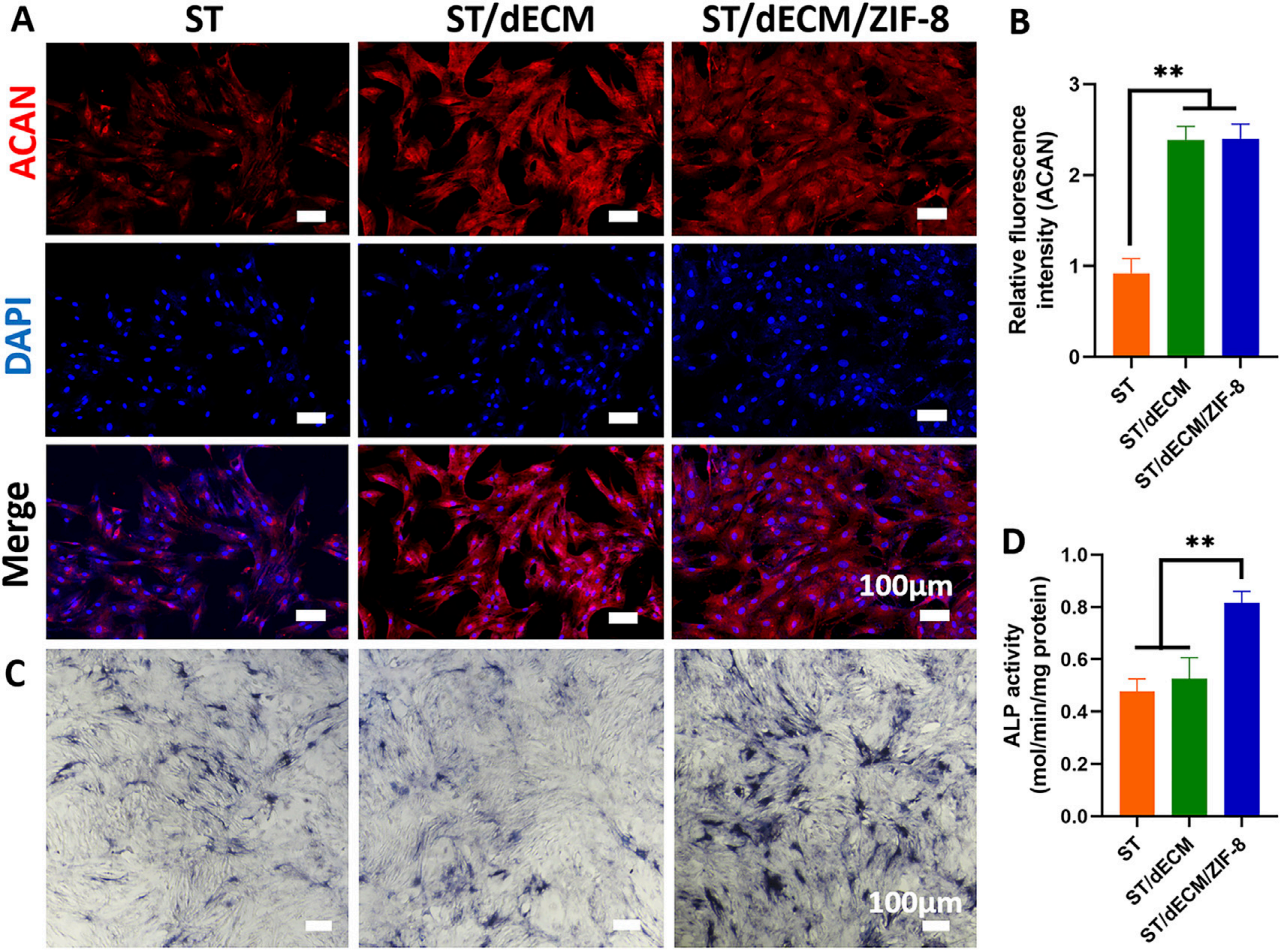
Effects of co-culture with different adhesives on ACAN protein expression and osteogenic differentiation media containing different adhesive extract solutions on ALP staining results in rat BMSCs. **(A)** Expression of ACAN protein in rat BMSCs co-cultured with different adhesives (scale bar = 100 μm). **(B)** Semi-quantitative analysis of fluorescence intensity for ACAN protein expression in rat BMSCs co-cultured with different adhesives (n = 3). **(C)** ALP staining results (scale bar = 100 μm). **(D)** Analysis of ALP activity assay results (n = 3).

After 14 days of co-culture of rat BMSCs with adhesives containing different formulations in chondrogenic induction medium, the expression levels of chondrogenic-related genes ACAN, and COL2 were significantly upregulated in both the ST/dECM and ST/dECM/ZIF-8 groups compared to the ST group (Figures 4C,D). Furthermore, immunofluorescence staining revealed that the protein expression levels of ACAN in the ST/dECM and ST/dECM/ZIF-8 groups were also significantly higher than in the ST group (Figures 5A,B). These results indicated that the addition of decellularized extracellular matrix to the adhesives promoted chondrogenic differentiation of rat BMSCs *in vitro*.

After 14 days of co-culture of rat BMSCs in osteogenic induction medium containing adhesive extracts with different formulations, the expression levels of osteogenesis-related genes RUNX2, and OCN were significantly higher in the ST/dECM/ZIF-8 group compared to the ST and ST/dECM groups (Figures 4E,F). After 14 days of co-culture in osteogenic induction medium, ALP staining results demonstrated a significant increase in ALP staining intensity in the ST/dECM/ZIF-8 group compared to the ST and ST/dECM groups, indicating a stronger osteogenic effect (Figure 5C). Additionally, semi-quantitative analysis of ALP activity assays further confirmed that the ST/dECM/ZIF-8 group had a more pronounced promoting effect on the osteogenic differentiation of rat BMSCs (Figure 5D).

### 3.6 Effects of adhesives with different formulations on the tissue compatibility of major organs and inflammatory microenvironment at the rotator cuff enthesis

Eight weeks after implantation of adhesives with different formulations, H&E staining revealed no significant damage to the heart, liver, spleen, lungs, or kidneys in rats (Supplementary Figure S3). These results indicated that the adhesives possessed good tissue compatibility with major organs.

**FIGURE 6:**
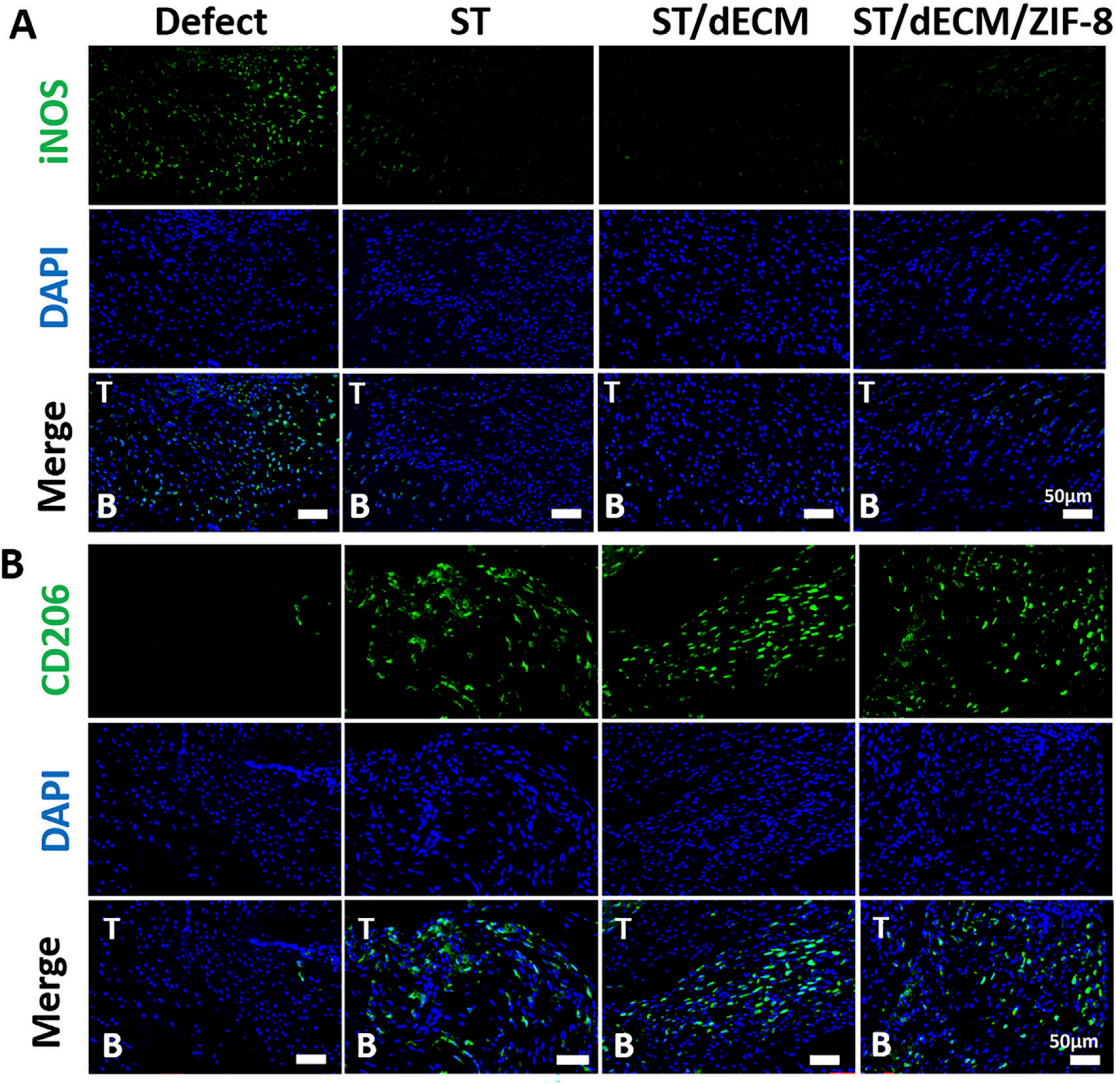
Representative immunofluorescence saining of macrophage polarization at the tendon-to-bone interface in rats from different groups at 8 weeks after surgery. **(A)** The iNOS (green) staining results show the distribution of M1-type macrophages (scale bar = 50 μm). **(B)** The CD206 (green) staining results show the distribution of M2-type macrophages (scale bar = 50 μm). T, tendon; B, bone.

To investigate the inflammatory response at the tendon-to-bone interface following rotator cuff repair, we performed iNOS and CD206 immunofluorescence staining on tissue sections collected 8 weeks post-surgery. Cells with high iNOS expression are indicative of M1-type macrophages, while those with high CD206 expression represent M2-type macrophages. INOS staining at 8 weeks post-operation revealed a high number of M1 macrophages at the enthesis in the Defect group, whereas the number of M1 macrophages was significantly reduced in the ST, ST/dECM, and ST/dECM/ZIF-8 groups (Figure 6A). CD206 staining demonstrated that the ST, ST/dECM, and ST/dECM/ZIF-8 groups had a significantly higher number of M2 macrophages at the tendon-to-bone interface compared to the Defect group (Figure 6B). These findings suggested that tannic acid in the adhesive might exert a strong immunomodulatory effect on the postoperative inflammatory response by promoting the activation of M2 macrophages and suppressing the excessive accumulation of M1 macrophages.

### 3.7 Histological effects of adhesives with different formulations on rotator cuff tendon-to-bone healing

**FIGURE 7:**
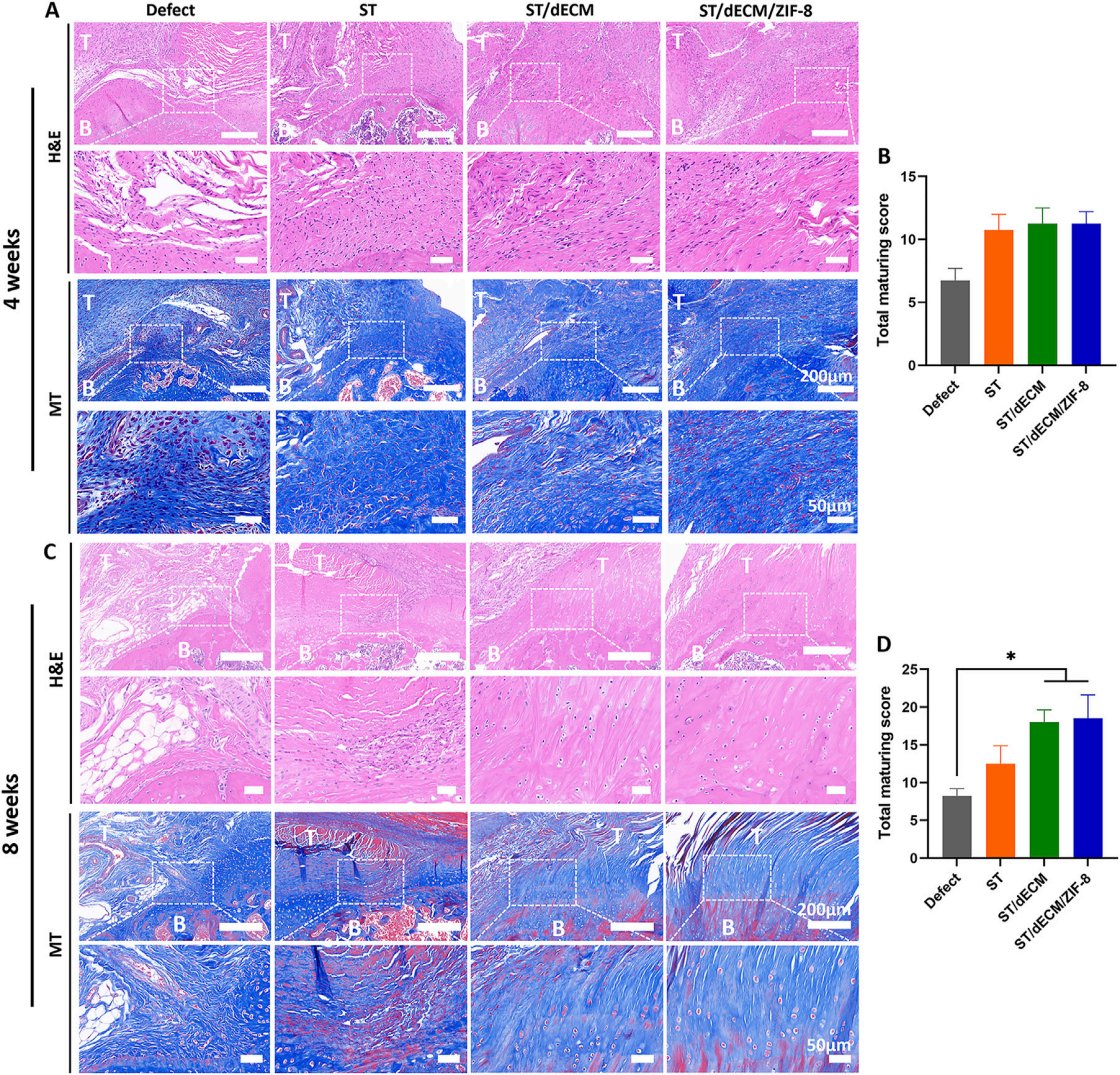
The histological staining and scores of different groups at 4 and 8 weeks after rat rotator cuff repair surgery. **(A)** The representative H&E and Masson’s Trichrome (MT) staining of tendon-to-bone interface results at 4 weeks postoperatively. **(B)** The total maturing score of tendon-to-bone interface at 4 weeks postoperatively (n = 4). **(C)** The representative H&E and Masson’s Trichrome staining of tendon-to-bone interface results at 8 weeks postoperatively. **(D)** The total maturing score of tendon-to-bone interface at 8 weeks postoperatively (n = 4). T, tendon; B, bone. The scale bars for the H&E staining and Masson’s trichrome staining are consistent.

Histological evaluation of the tendon-to-bone interface at 4 weeks post-surgery revealed that although the ST, ST/dECM, and ST/dECM/ZIF-8 groups exhibited higher histological scores than the Defect group (ST vs. Defect: P = 0.169; ST/dECM vs. Defect: P = 0.083; ST/dECM/ZIF-8 vs. Defect: P = 0.054), no statistically significant differences were observed between groups (Figures 7A,B). At 8 weeks postoperatively, histological assessment of the tendon-to-bone interface at this time point showed significantly higher scores in the ST/dECM and ST/dECM/ZIF-8 groups compared to the Defect group. Although the scores were also higher than those in the ST group, the differences were not statistically significant (Figures 7C,D).

**FIGURE 8:**
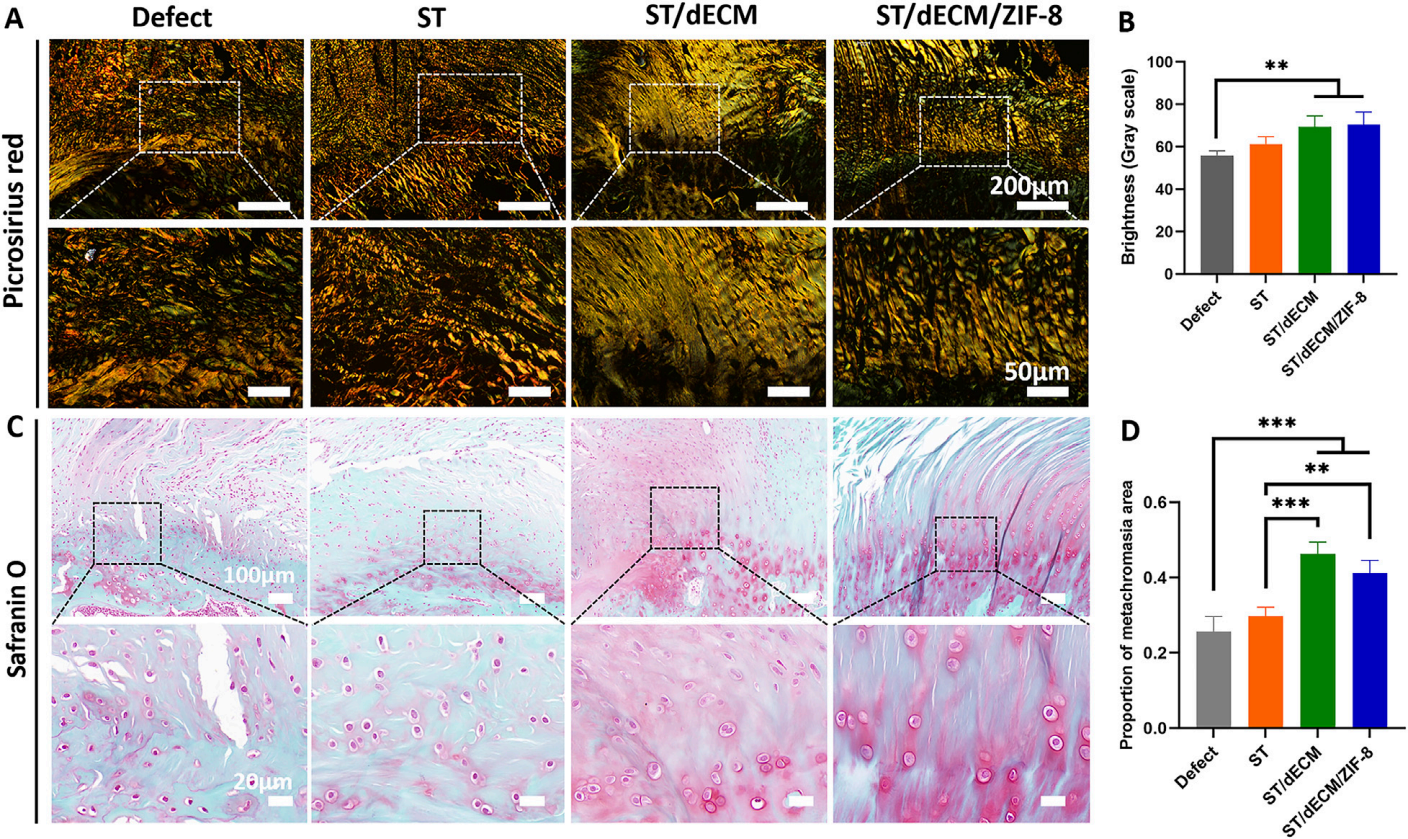
Picrosirius red and safranin O staining of rotator cuff entheses in rats 8 weeks after surgery. **(A)** Picrosirius red staining of tendon-bone interface. **(B)** Grayscale quantification of picrosirius red staining (n = 4). **(C)** Safranin O staining of tendon-bone interfaces. **(D)** Semi-quantitative analysis of Safranin O staining intensity (n = 4).

Polarized light microscopy with picrosirius red staining was used to distinguish collagen types: type I collagen appeared orange or red, while type III collagen appeared green. At 8 weeks postoperatively, the fiber orientation became aligned, particularly in the ST/dECM and ST/dECM/ZIF-8 groups, which showed notably improved structural organization (Figure 8A). Semi-quantitative analysis at 8 weeks post-surgery showed that the ST/dECM and ST/dECM/ZIF-8 groups had significantly higher values than the Defect group, and the ST group also showed some improvement (Figure 8B). These results confirmed that adhesive implantation promoted type I collagen deposition and organized fiber remodeling, thereby enhancing the quality of tendon repair.

For safranin O staining, cartilage appears red. At 8 weeks post-surgery, the ST/dECM and ST/dECM/ZIF-8 groups displayed larger red-stained areas at the tendon-to-bone interface compared to the Defect and ST groups, indicating that adhesives containing dECM promoted greater cartilage formation at the repair site (Figures 8C,D). These findings suggest that the addition of dECM enhanced cartilage regeneration at the tendon-to-bone interface.

### 3.8 Effects of adhesives with different formulations on rotator cuff bone tunnel healing

**FIGURE 9:**
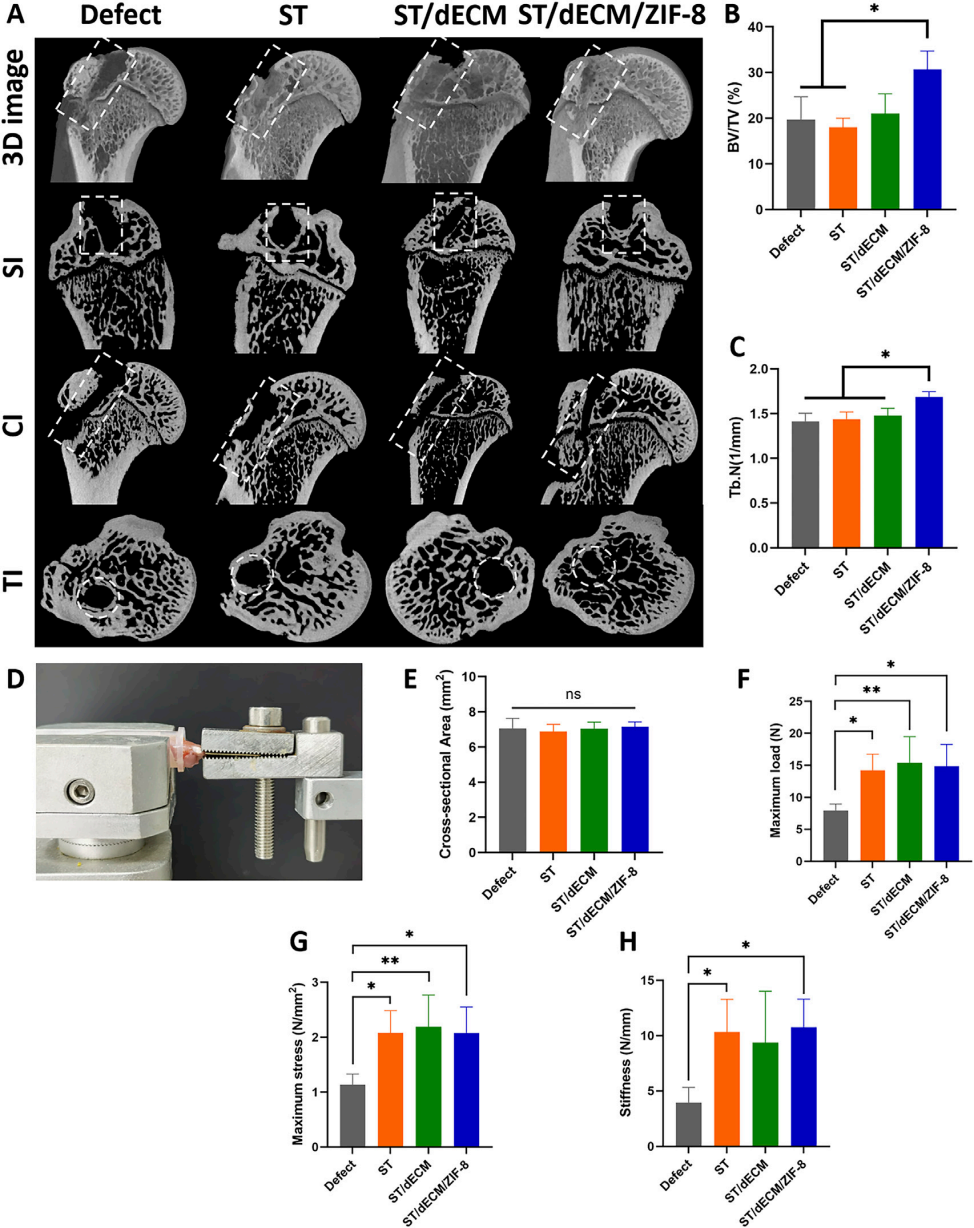
Healing status of humeral bone tunnels and mechanical testing of humerus-supraspinatus units in rats from different groups at 8 weeks after rotator cuff repair. **(A)** Representative micro-CT scan images showing 3D reconstruction, sagittal, coronal, and transverse views of the rat humerus; bone tunnel areas are outlined with white dashed lines. **(B)** Analysis of BV/TV results for regenerated bone tissue (n = 3). **(C)** Analysis of Tb.N results for regenerated bone tissue (n = 3). SI, sagittal images; CI, coronal images; TI, transverse images. **(D)** The mechanical testing platform. **(E)** The cross-sectional area of the tendon-to-bone interface (n = 5). **(F)** The maximum load of tendon-to-bone interface (n = 5). **(G)** The maximum stress of tendon-to-bone interface (n = 5). **(H)** The stiffness of tendon-to-bone interface (n = 5).

The regeneration of the humeral bone tunnel in rats was evaluated using micro-CT at 8 weeks post-surgery (Figure 9A). The bone volume fraction (BV/TV) in the ST/dECM/ZIF-8 group was 30.67% ± 4.04%, which was significantly higher than that in the Defect group (19.67% ± 5.03%) and the ST group (18.00% ± 2.00%). Although the BV/TV was not significantly different from the ST/dECM group (21.00% ± 4.36%), it still showed a noticeable increase (Figure 9B). The trabecular number (Tb.N) in the ST/dECM/ZIF-8 group was 1.69 ± 0.06 1/mm, significantly higher than that in the Defect group (1.41 ± 0.09 1/mm), ST group (1.44 ± 0.08 1/mm), and ST/dECM group (1.48 ± 0.08 1/mm) (Figure 9C). These results indicated that the ST/dECM/ZIF-8 group significantly promoted bone tunnel healing at 8 weeks postoperatively compared to the Defect and ST groups.

### 3.9 Effects of adhesives with different formulations on the biomechanical properties of rotator cuff tendon-to-bone healing

No obvious gross morphological differences were observed among the humerus-supraspinatus complexes in each group at 8 weeks post-surgery (Supplementary Figure S4). Biomechanical testing of the humerus-supraspinatus units was performed using the platform (Figure 9D), following previously reported tensile loading directions (He et al., 2021; Chae et al., 2021). There were no significant differences in the cross-sectional area at the tendon-bone insertion site among the groups (Figure 9E). The maximum load to failure was significantly higher in the ST (14.25 ± 2.50 N), ST/dECM (15.38 ± 4.10 N), and ST/dECM/ZIF-8 (14.83 ± 3.43 N) groups compared to the Defect group (7.91 ± 1.04 N), although no significant differences were observed among the three adhesive groups (Figure 9F). Similarly, the maximum stress in the ST (2.08 ± 0.41 N/mm^2^), ST/dECM (2.19 ± 0.41 N/mm^2^), and ST/dECM/ZIF-8 (2.07 ± 0.47 N/mm^2^) groups was significantly higher than in the Defect group, but again, no significant differences were detected among the three adhesive-treated groups (Figure 9G). The maximum stiffness in the ST (10.33 ± 2.95 N/mm) and ST/dECM/ZIF-8 (10.74 ± 2.55 N/mm) groups was significantly higher than in the Defect group (3.96 ± 1.35 N/mm). Although the ST/dECM group (9.36 ± 4.64 N/mm) showed increased stiffness as well, no significant differences were found among the three adhesive-treated groups (Figure 9H). These results indicated that all three adhesive formulations significantly improved the biomechanical strength of tendon-to-bone healing in the rotator cuff repair model. The enhancement in mechanical performance might be attributed to the increased adhesive strength provided by the combination of silk fibroin and tannic acid used in the adhesives.

## 4 Discussion

In this study, an adhesive system was developed based on dECM derived from the porcine intervertebral disc annulus fibrosus. This system integrates multiple functional components, including silk fibroin, tannic acid, dECM, and ZIF-8. Silk fibroin and tannic acid serve as the base matrix of the adhesive, providing strong tissue adhesion. The dECM, derived from the porcine annulus fibrosus, contains fibrocartilaginous components that can promote the chondrogenic differentiation of BMSCs and facilitate cartilage regeneration. ZIF-8 is incorporated into the adhesive through metal coordination and plays a role in enhancing the osteogenic differentiation of BMSCs, thus promoting bone regeneration.

This study makes several unique contributions. Firstly, while traditional adhesives often focus solely on mechanical performance, this study emphasizes a dual strategy of enhancing adhesion and promoting tissue regeneration through inflammation modulation and biomimicry. Secondly, the rational selection of materials with strong clinical translation potential significantly improves the feasibility of future clinical applications. Lastly, this work expands the application of annulus fibrosus-derived dECM to tendon-to-bone interface repair, offering new insights into the development of tissue-specific biomaterials.

Silk fibroin and tannic acid, as the base materials of the adhesive, were positioned between the tendon and bone tissue during rotator cuff repair, significantly improving the biomechanical strength of the tendon-to-bone interface. According to previous reports, hydrogels prepared using porcine cartilage-derived dECM do not induce significant immune rejection and exhibit good cellular and tissue compatibility when implanted in rats or mice (Zeng et al., 2022; Li et al., 2024). In this study, we did not directly use tannic acid and dECM to form a hydrogel, as the combination of these two components showed poor gelation properties and insufficient tissue adhesion. Therefore, we introduced a mixture of silk fibroin and dECM, which was further combined with tannic acid to form a hydrogel with strong adhesion under wet conditions.

Although adhesives based on silk fibroin and tannic acid have been previously used in treating bone defects, root caries, and hemostasis (Zou et al., 2023; Li et al., 2025), this study is the first to explore their application in tendon-to-bone interface repair. Tannic acid forms a hydrogel with silk fibroin through hydrogen bonding between its polyphenol groups and the amide groups of silk fibroin. The tissue adhesive strength of this system was comparable to previously reported adhesives (Zou et al., 2023; Luo et al., 2020). Based on the results of *in vitro* tissue adhesion tests, we selected a formulation combining the silk fibroin-tannic acid matrix with a high concentration of dECM solution. Given the concentration-dependent osteogenic promotion of ZIF-8 and the potential cytotoxicity at higher concentrations, we selected ZIF-8 concentrations of 0.5 wt% and 1 wt% for incorporation into the adhesive system, based on previous reports (Tang et al., 2024). Wet adhesion testing revealed that increasing ZIF-8 content led to a decrease in tissue adhesion strength. This may be due to a larger proportion of catechol groups forming metal-catechol coordination bonds with ZIF-8 nanoparticles, which in turn reduces their interaction with tissue proteins (Filippidi et al., 2017).

Extracts from different adhesive formulations supported good cell viability and proliferation *in vitro*. Adhesives containing porcine annulus fibrosus dECM significantly promoted chondrogenic differentiation of rat BMSCs, whereas the inclusion of ZIF-8 enhanced osteogenic differentiation. In terms of extract concentration, we referred to previous studies and prepared a system by mixing 5 mg of lyophilized adhesive powder with 25 mL of culture medium, which facilitated rapid release of active components (Zou et al., 2023; Guo et al., 2024). At this concentration, BMSCs showed good cell viability and proliferation. For chondrogenic differentiation of rat BMSCs, cells were directly co-cultured with the adhesive, allowing direct contact between dECM components to better induce differentiation. The inclusion of porcine annulus fibrosus dECM significantly promoted chondrogenic differentiation of BMSCs. For osteogenic differentiation, the adhesive extract containing ZIF-8 significantly enhanced BMSC differentiation toward osteogenic lineages. Previous studies have also demonstrated that ZIF-8 incorporated into scaffolds or hydrogels promotes osteogenic differentiation *in vitro* and supports bone tissue regeneration *in vivo* (Tang et al., 2024; Qin et al., 2025; Zhao et al., 2023).

The adhesives with different formulations were evaluated *in vivo* using a rat model of rotator cuff injury repair. The dECM-based adhesive not only exhibited excellent tissue compatibility but also significantly improved tissue maturation and the orderly arrangement of collagen fibers at the tendon-to-bone interface after 8 weeks postoperatively. Due to the high similarity in both composition and structure between the annulus fibrosus-derived dECM and the native rotator cuff enthesis, the inclusion of dECM in the adhesive effectively promoted fibrocartilage regeneration at the tendon-to-bone junction. Furthermore, the incorporation of ZIF-8 further enhanced the healing of the bone tunnel within the rotator cuff.

Tannic acid, owing to its anti-inflammatory properties, modulated the inflammatory microenvironment at the tendon-to-bone interface. At 8 weeks postoperatively, adhesives composed of silk fibroin and tannic acid increased the number of M2 macrophages while reducing M1 macrophages at the enthesis compared to the repair-only group, indicating a positive effect on tendon-to-bone healing. Both ST/dECM/ZIF-8 and ST/dECM groups significantly improved the overall maturity of the tendon-to-bone interface, although no significant differences were observed between them. This may be due to limitations in the homogenous material design used in the current study, which fails to replicate the gradient mineralization structure of the native tendon-to-bone junction. These findings suggest that constructing multilayer structures with spatial heterogeneity may be more effective in recreating the complex biomechanical and biological microenvironment of the interface.

Micro-CT analysis showed that the ST/dECM/ZIF-8 group had higher BV/TV and Tb.N compared to the other three groups, indicating that the inclusion of ZIF-8 in the adhesive promoted bone tunnel healing. In clinical practice, suture anchors are commonly used for rotator cuff repair. Promoting bone regeneration may reduce the risk of anchor loosening and subsequent surgical failure. At 8 weeks post-surgery, biomechanical testing of the humerus-supraspinatus tendon complex showed that all adhesive-treated groups had significantly higher biomechanical strength compared to the defect group. However, there were no significant differences among the three adhesive groups, suggesting that the increased adhesion at the tendon-to-bone interface was primarily due to the contribution of the silk fibroin-tannic acid matrix.

There are several limitations of this study. The rat model employed was based on acute injury, whereas human rotator cuff injuries typically follow a chronic progression. Therefore, the findings may not be directly extrapolatable to human patients. In addition, only male subjects were used, which may introduce bias due to the absence of sex-based analysis. Lastly, although the adhesive demonstrated wet tissue adhesion, its strength under dynamic, wet physiological conditions still falls short of that found at the native tendon-to-bone interface. Further research is needed to improve adhesion performance under such conditions.

Despite the progress made in this study, several areas merit further investigation. Future work could explore the design of gradient adhesive structures with spatiotemporal heterogeneity to better mimic the native transition from soft tissue to calcified cartilage to bone, potentially enhancing biomechanical integration at the tendon-to-bone interface. Long-term evaluations in large animal models are needed to assess the adhesive’s regenerative effects and integration with host tissue. Furthermore, multi-omics sequencing approaches could be employed to investigate how each component of the adhesive regulates intracellular signaling pathways to influence macrophage polarization and BMSC differentiation toward different lineages.

## 5 Conclusion

The ST/dECM/ZIF-8 adhesive demonstrated strong tissue adhesion under wet conditions. Bioactive components within the decellularized porcine intervertebral disc annulus fibrosus extracellular matrix promoted the chondrogenic differentiation of BMSCs. Meanwhile, the incorporation of ZIF-8 upregulated the expression of genes associated with osteogenic differentiation in BMSCs. ST/dECM/ZIF-8 adhesive led to significant improvements at the tendon-to-bone interface: the local inflammatory microenvironment was effectively alleviated, the fibrocartilage layer was markedly thickened, collagen fibers were more orderly arranged, and bone density within the bone tunnel region was increased. Compared to the suture-only group, the humerus-supraspinatus tendon complex in the adhesive-treated group exhibited significant enhancements in mechanical properties, including ultimate load, ultimate stress, and maximum stiffness. These findings indicate that the ST/dECM/ZIF-8 adhesive holds promise for enhancing the mechanical performance of the repair site and promoting tissue regeneration.

## Data Availability

The original contributions presented in the study are included in the article/Supplementary Material, further inquiries can be directed to the corresponding authors.

## References

[B1] Bai L. Han Q. Meng Z. Chen B. Qu X. Xu M. (2022). Bioprinted living tissue constructs with layer-specific, growth factor-loaded microspheres for improved enthesis healing of a rotator cuff. Acta Biomater. 154, 275–289. 10.1016/j.actbio.2022.10.058 36328126

[B2] Chae S. Sun Y. Choi Y. Ha D. Cho D. (2021). 3D cell-printing of tendon-bone interface using tissue-derived extracellular matrix bioinks for chronic rotator cuff repair. Biofabrication 13, 035005. 10.1088/1758-5090/abd159 33285539

[B3] Choi S. Lee M. Kim M. Bae Y. Park J. Cho S. (2024). Durable muscle extracellular matrix engineered with adhesive phenolic moieties for effective skeletal muscle regeneration in muscle atrophy. Adv. Healthc. Mater. 13, 2401826. 10.1002/adhm.202401826 39420690 PMC11670296

[B4] Deprés-tremblay G. Chevrier A. Snow M. Hurtig M. B. Rodeo S. Buschmann M. D. (2016). Rotator cuff repair: a review of surgical techniques, animal models, and new technologies under development. J. Shoulder Elb. Surg. 25, 2078–2085. 10.1016/j.jse.2016.06.009 27554609

[B5] Dimitrios C. Christopher C. Paul K. Neal L. M. (2019). How does surgery compare to sham surgery or physiotherapy as a treatment for tendinopathy? A systematic review of randomised trials. BMJ Open Sport Exerc. Med. 5, e000528. 10.1136/bmjsem-2019-000528 PMC6539146 31191975

[B6] Duan B. Hockaday L. Das S. Xu C. Butcher J. (2015). Comparison of mesenchymal stem cell source differentiation toward human pediatric aortic valve interstitial cells within 3D engineered matrices. Tissue Eng. Part C Methods. 21, 795–807. 10.1089/ten.tec.2014.0589 25594437 PMC4523011

[B7] Filippidi E. Cristiani T. Eisenbach C. Waite J. Israelachvili J. Ahn B. (2017). Toughening elastomers using mussel-inspired iron-catechol complexes. Science 358, 502–505. 10.1126/science.aao0350 29074770 PMC5676464

[B8] Gao X. Dai Q. Yao L. Dong H. Li Q. Cao X. (2020). A medical adhesive used in a wet environment by blending tannic acid and silk fibroin. Biomater. Sci. 8, 2694–2701. 10.1039/d0bm00322k 32267256

[B9] Guo Z. Li M. Sun N. Song T. Wang C. (2024). A multifunctional bioadhesive for bone augmentation: tissue integration combined with antioxidant and antibacterial effects. ACS Appl. Mater. & Interfaces 16 (48), 65890–65906. 10.1021/acsami.4c14863 39574236

[B10] He S. Ning L. Yao X. Hu R. Cui J. Zhang Y. (2021). Hierarchically demineralized cortical bone combined with stem cell-derived extracellular matrix for regeneration of the tendon-bone interface. Am. J. Sports Med. 49, 1323–1332. 10.1177/0363546521994511 33667131

[B11] Ide J. Kikukawa K. Hirose J. Iyama K. Sakamoto H. Mizuta H. (2009). The effects of fibroblast growth factor-2 on rotator cuff reconstruction with acellular dermal matrix grafts. Arthroscopy 25, 608–616. 10.1016/j.arthro.2008.11.011 19501290

[B12] Jiang X. Kong Y. Kuss M. Weisenburger J. Haider H. Harms R. (2022). 3D bioprinting of multilayered scaffolds with spatially differentiated ADMSCs for rotator cuff tendon-to-bone interface regeneration. Appl. Mater. Today. 27, 101510. 10.1016/j.apmt.2022.101510

[B13] Jost B. Fau Z. M. Pfirrmann C. W. A. Fau P. C. Gerber C. Gerber C. (2006). Long-term outcome after structural failure of rotator cuff repairs. J. Bone Jt. Surg. 88, 472–479. 10.2106/JBJS.E.00003 16510810

[B14] Killian M. L. Cavinatto L. Shah S. A. Sato E. J. Havlioglu N. Galatz L. M. (2014). The effects of chronic unloading and gap formation on tendon-to-bone healing in a rat model of massive rotator cuff tears. J. Orthop. Res. 32, 439–447. 10.1002/jor.22519 24243733 PMC3900302

[B15] Kim E. Jung J. Yoon S. Park W. (2023). Eco-friendly silk fibroin/tannic acid coacervates for humid and underwater wood adhesives. J. Colloid Interface Sci. 632, 151–160. 10.1016/j.jcis.2022.11.017 36413941

[B16] Kong Y. Shi W. Zhang D. Jiang X. Kuss M. Liu B. (2021). Injectable, antioxidative, and neurotrophic factor-deliverable hydrogel for peripheral nerve regeneration and neuropathic pain relief. Appl. Mater. Today. 24, 101090. 10.1016/j.apmt.2021.101090

[B17] Li J. Jiang B. Zhang J. Wu N. Fan Z. Chen Y. (2024). Cartilage decellularized extracellular matrix-based hydrogel with enhanced tissue adhesion and promoted chondrogenesis for cartilage tissue engineering. ACS Appl. Polym. Mater. 6, 4394–4408. 10.1021/acsapm.3c01733

[B18] Li M. Su Z. Zhu J. Zhen L. Huang X. Luo J. (2025). Clinically oriented oral environment-triggered underwater adhesives for root caries treatment through dentinal tubule occlusion and remineralization. ACS Appl. Mater. Interfaces. 17, 16576–16589. 10.1021/acsami.4c20161 40052410

[B19] Liu B. Alimi O. A. Wang Y. Kong Y. Kuss M. A. Krishnan M. (2024). Differentiated mesenchymal stem cells-derived exosomes immobilized in decellularized sciatic nerve hydrogels for peripheral nerve repair. J. Control. Release. 368, 24–41. 10.1016/j.jconrel.2024.02.019 38367864 PMC11411504

[B20] Liu C. Ge X. Li Y. (2024). Repair of annulus fibrosus defects using decellularized annulus fibrosus matrix/chitosan hybrid hydrogels. J. Orthop. Surg. Res. 19, 535. 10.1186/s13018-024-05017-y 39223621 PMC11370001

[B21] Liu C. Li Y. Zhang Y. Xu H. (2022). The experimental study of regeneration of annulus fibrosus using decellularized annulus fibrosus matrix/poly(ether carbonate urethane)urea-blended fibrous scaffolds with varying elastic moduli. J. Biomed. Mater. Res. A 110, 991–1003. 10.1002/jbm.a.37347 34918475

[B22] Liu C. Liang X. Yu Z. Quanlai Z. Xu H. (2020). Regeneration of annulus fibrosus tissue using a DAFM/PECUU-blended electrospun scaffold. J. Biomater. Sci. Polym. Ed. 31, 2347–2361. 10.1080/09205063.2020.1812038 32885742

[B23] Luo J. Yang J. Zheng X. Ke Y. Chen H. Tan J. (2020). A highly stretchable, real-time self-healable hydrogel adhesive matrix for tissue patches and flexible electronics. Adv. Healthc. Mater. 9, 1901423. 10.1002/adhm.201901423 31945276

[B24] Maghsoudi M. Aghdam R. Asbagh R. Moghaddaszadeh A. Ghaee S. Tafti L. (2024). 3D-printing of alginate/gelatin scaffold loading tannic acid@ZIF-8 for wound healing: *in vitro* and *in vivo* studies. Int. J. Biol. Macromol. 265, 130744. 10.1016/j.ijbiomac.2024.130744 38493825

[B25] Nam H. G. Nam M. G. Yoo P. J. Kim J. H. (2019). Hydrogen bonding-based strongly adhesive coacervate hydrogels synthesized using poly(N-vinylpyrrolidone) and tannic acid. Soft Matter 15, 785–791. 10.1039/c8sm02144a 30638244

[B26] Ogawa R. Mizuno H. Watanabe A. Migita M. Shimada T. Hyakusoku H. (2004). Osteogenic and chondrogenic differentiation by adipose-derived stem cells harvested from GFP transgenic mice. Biochem. Biophys. Res. Commun. 313, 871–877. 10.1016/j.bbrc.2003.12.017 14706623

[B27] Olvera D. Sathy B. N. Kelly D. J. (2020). Spatial presentation of tissue-specific extracellular matrix components along electrospun scaffolds for tissue engineering the bone-ligament interface. ACS Biomater. Sci. Eng. 6, 5145–5161. 10.1021/acsbiomaterials.0c00337 33455265

[B28] Qin K. Huang X. Wang S. Liang Z. Fan Z. (2025). 3D-printed *in situ* growth of bilayer MOF hydrogels for accelerated osteochondral defect repair. Adv. Healthc. Mater. 14, 2403840. 10.1002/adhm.202403840 39552270

[B29] Rockwood D. N. Preda R. C. Yücel T. Wang X. Lovett M. L. Kaplan D. L. (2011). Materials fabrication from Bombyx mori silk fibroin. Nat. Protoc. 6, 1612–1631. 10.1038/nprot.2011.379 21959241 PMC3808976

[B30] Shi W. Kong Y. Su M. A. Kuss X. Jiang X. Li J. (2021). Tannic acid-inspired, self-healing, and dual stimuli responsive dynamic hydrogel with potent antibacterial and anti-oxidative properties. J. Mat. Chem. B 9, 7182–7195. 10.1039/d1tb00156f 33651063

[B31] Song W. Guo Y. Liu W. Yao Y. Zhang X. Cai Z. (2024). Circadian rhythm-regulated ADSC-derived sEVs and a triphasic microneedle delivery system to enhance tendon-to-bone healing. Adv. Mater. 36, 2408255. 10.1002/adma.202408255 39120049

[B32] Tang H. Yu Y. Zhan Y. Chai Y. Zheng Y. Liu D. (2024). Zeolite imidazolate framework-8 in bone regeneration: a systematic review. J. Control. Release. 365, 558–582. 10.1016/j.jconrel.2023.11.049 38042375

[B33] Tang Y. Chen C. Liu F. Xie S. Qu J. Li M. (2020). Structure and ingredient-based biomimetic scaffolds combining with autologous bone marrow-derived mesenchymal stem cell sheets for bone-tendon healing. Biomaterials 241, 119837. 10.1016/j.biomaterials.2020.119837 32109704

[B34] Van Buren J. P. Robinson W. B. (1969). Formation of complexes between protein and tannic acid. J. Agric. Food Chem. 17, 772–777. 10.1021/jf60164a003

[B35] Wang J. Chen X. Chen Y. Qin F. Yang H. (2022). Pharmacological effects and mechanisms of tannic acid. Biomed. Pharmacother. 154, 113561. 10.1016/j.biopha.2022.113561 36029537

[B36] Wang R. He X. Su S. Bai J. Liu H. Zhou F. (2024). Multifunctional tannic acid-based nanocomposite methacrylated silk fibroin hydrogel with the ability to scavenge reactive oxygen species and reduce inflammation for bone regeneration. Int. J. Biol. Macromol. 266, 131357. 10.1016/j.ijbiomac.2024.131357 38580010

[B37] Yakacki C. M. Poukalova M. Guldberg R. E. Lin A. Saing S. Gillogly K. (2010). The effect of the trabecular microstructure on the pullout strength of suture anchors. J. Biomech. 43, 1953–1959. 10.1016/j.jbiomech.2010.03.013 20399431 PMC2900467

[B38] Yang B. Yao H. Yao J. Chen C. Shi J. (2022). Construction of a two-dimensional artificial antioxidase for nanocatalytic rheumatoid arthritis treatment. Nat. Commun. 13, 1988. 10.1038/s41467-022-29735-1 35418125 PMC9008001

[B39] Yuan M. Yan S. Liu H. Kundu S. C. Cai Y. Yao J. (2019). Performance of water-immiscible silk fibroin based hydrogel as underwater biomedical adhesive. Fiber. Polym. 20, 2032–2041. 10.1007/s12221-019-1206-9

[B40] Zeng J. Huang L. Xiong Q. Li C. Wu Y. Huang H. (2022). Injectable decellularized cartilage matrix hydrogel encapsulating urine-derived stem cells for immunomodulatory and cartilage defect regeneration. npj Regen. Med. 7, 75. 10.1038/s41536-022-00269-w 36550127 PMC9780205

[B41] Zhang J. Hao R. Huang L. Yao J. Chen Z. Shao Z. (2011). Self-assembly of a peptide amphiphile based on hydrolysed Bombyx mori silk fibroin. Chem. Commun. 47, 10296–10298. 10.1039/c1cc12633d 21858302

[B42] Zhang X. Bogdanowicz D. Erisken C. Lee N. M. Lu H. H. (2012). Biomimetic scaffold design for functional and integrative tendon repair. J. Shoulder Elb. Surg. 21, 266–277. 10.1016/j.jse.2011.11.016 PMC3260004 22244070

[B43] Zhang X. Zhai H. Zhu X. Geng Y. Zhang J. Cui Y. (2024). Polyphenol-mediated adhesive and anti-inflammatory double-network hydrogels for repairing postoperative intervertebral disc defects. ACS Appl. Mater. Interfaces 16, 53541–53554. 10.1021/acsami.4c11901 39344595

[B44] Zhao C. Shu J. Yu Y. Zhu Z. (2023). Metal-organic frameworks functionalized biomaterials for promoting bone repair. Mater. Today bio. 21, 100717. 10.1016/j.mtbio.2023.100717 PMC10401359 37545559

[B45] Zhao L. Luo J. Cui J. Li X. Hu R. Xie X. (2024). Tannic acid-modified decellularized tendon scaffold with antioxidant and anti-inflammatory activities for tendon regeneration. ACS Appl. Mater. Interfaces. 16 (13), 15879–15892. 10.1021/acsami.3c19019 38529805

[B46] Zhou K. Mousavi Z. Luo S. Phatanasri S. Chaemchuen F. Verpoort F. (2017). Characterization and properties of Zn/Co zeolitic imidazolate frameworks vs. ZIF-8 and ZIF-67. J. Mater. Chem. A 5, 952–957. 10.1039/c6ta07860e

[B47] Zou Y. Liang H. Wang B. Zhang Q. Su D. Lu S. Y. (2023). Precipitation-based silk fibroin fast gelling, highly adhesive, and magnetic nanocomposite hydrogel for repair of irregular bone defects. Adv. Funct. Mater. 33, 2302442. 10.1002/adfm.202302442

