# Supplementary material for: ZIF-8-loaded decellularized porcine annulus fibrosus bioadhesive enhances rotator cuff tendon-to-bone healing in a rat model
Source: Front Bioeng Biotechnol. 2025 Jul 22;13:1642818. doi: 10.3389/fbioe.2025.1642818 (PMC12322669; doi:10.3389/fbioe.2025.1642818)
Supplement: Supplementary file 1 [file Supplementaryfile1.docx]

Supplementary Material

1. **Supplementary materials and methods**
2. **Histocompatibility evaluation of adhesives with major organs**

At 8 weeks post-surgery, the heart, liver, spleen, lungs, and kidneys were harvested from rats in the ST, ST/dECM, and ST/dECM/ZIF-8 groups to assess whether the locally implanted hydrogels caused any adverse effects on major organ tissues. The collected tissues were subjected to fixation, dehydration, paraffin embedding, sectioning, and hematoxylin and eosin (H&E) staining.

1. **Supplementary figures and tables**

**
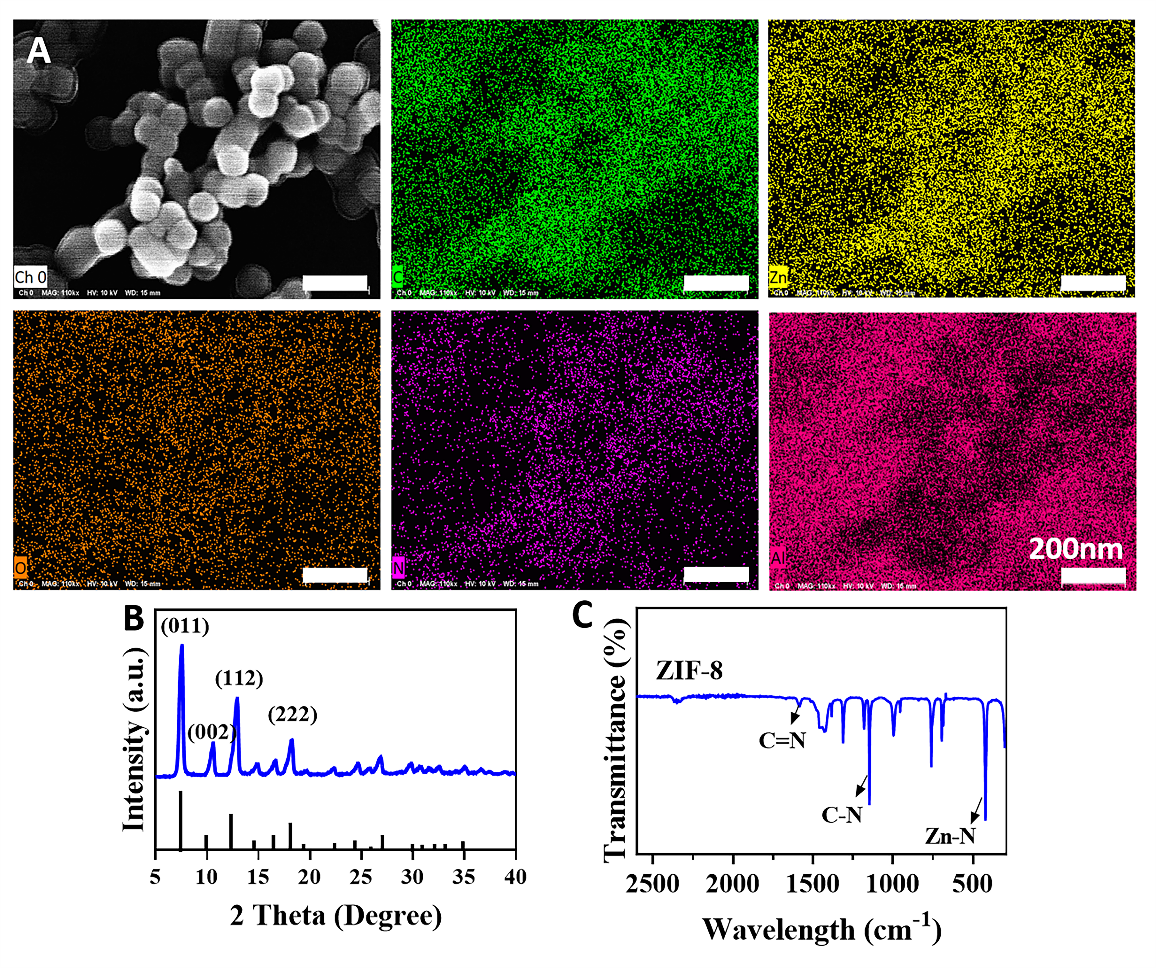
**

**Supplementary Figure 1. The SEM, EDX, XRD, and FTIR results of ZIF-8. (**A) The SEM and EDX results of ZIF-8. (B) The XRD result of ZIF-8. (C) The FTIR result of ZIF-8.

**
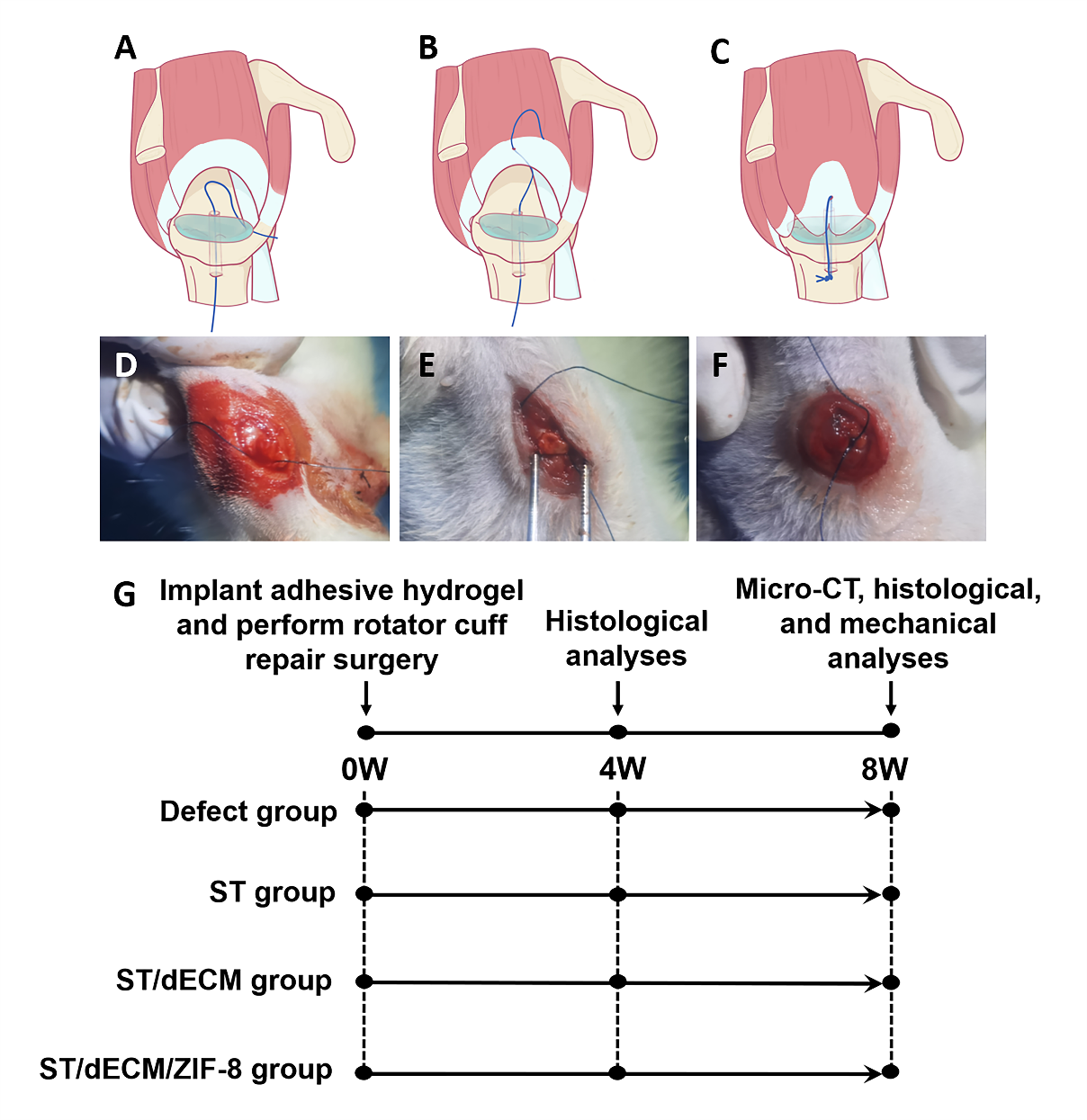
**

**Supplementary Figure 2. Images and schematics of adhesive implantation in a rotator cuff repair rat model.** (A, D) Establishment of a rotator cuff tear rat model. (B, E) Implantation of the adhesive. (C, F) Repair of rotator cuff tear. (G) Schematic diagram of the in vivo experimental protocol for rat rotator cuff injury repair with implantation of different adhesive hydrogels.

**
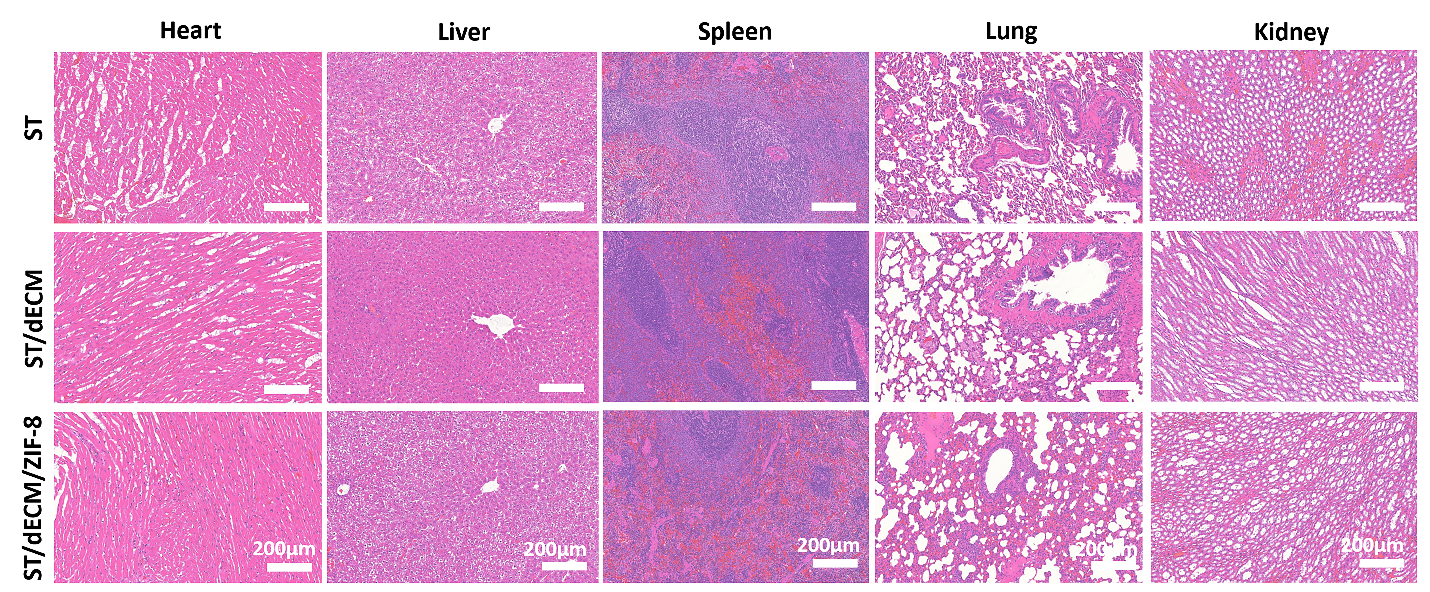
**

**Supplementary Figure 3. The H&E staining of heart, liver, spleen, lung, and kidney at 8 weeks after the implantation of different adhesive hydrogels into rat.**

**
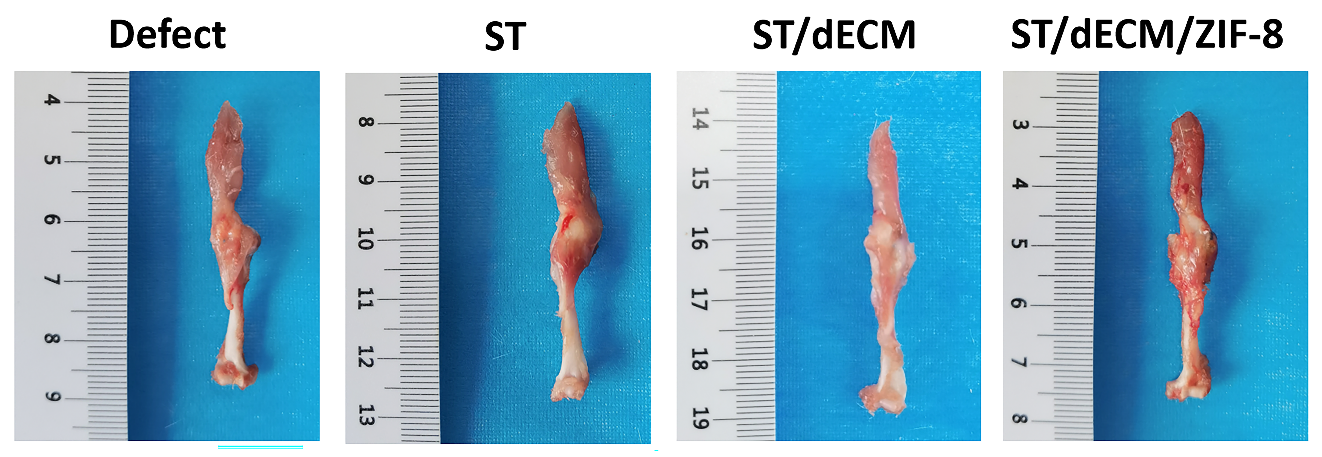
**

**Supplementary Figure 4. The harvested humerus-supraspinatus units with different study groups at 8 weeks after surgery.**

**Supplementary Table 1. Primer sequences for qPCR**

| Gene symbol | Primer sequences（5’→3’） |
| --- | --- |
| COL2 | F: GTGTCAAGGGTCACAGAGGTTAC  R: CGCTCTCACCCTTCACACCT |
| ACAN | F: GGTGTCACTTCCCAACTATCCAG  R: GGCTCGGTCAAAGTCCAGTG |
| RUNX2 | F: CAGTATGAGAGTAGGTGTCCCGC  R: AAGAGGGGTAAGACTGGTCATAGG |
| OCN | F: TGACAAAGCCTTCATGTCCAA  R: CTCCAAGTCCATTGTTGAGGTAG |
| GADPH | F: CTGGAGAAACCTGCCAAGTATG  R: GGTGGAAGAATGGGAGTTGCT |
